# Comparative chloroplast genome analyses of *Avena*: insights into evolutionary dynamics and phylogeny

**DOI:** 10.1186/s12870-020-02621-y

**Published:** 2020-09-02

**Authors:** Qing Liu, Xiaoyu Li, Mingzhi Li, Wenkui Xu, Trude Schwarzacher, John Seymour Heslop-Harrison

**Affiliations:** 1 Key Laboratory of Plant Resources Conservation and Sustainable Utilization / Guangdong Provincial Key Laboratory of Applied Botany, South China Botanical Garden, Chinese Academy of Sciences, Guangzhou, China; 2 Center for Conservation Biology, Core Botanical Gardens, Chinese Academy of Sciences, Guangzhou, China; 3 University of Chinese Academy of Sciences, Beijing, China; 4 Independent Researcher, Guangzhou, China; 5 Department of Genetics and Genome Biology, University of Leicester, Leicester, LE1 7RH UK

**Keywords:** *Avena*, Chloroplast genome, Evolution rate, Insertions/deletions, Intermolecular recombination, Phylogenomics, Single nucleotide polymorphisms, Tandem repeats

## Abstract

**Background:**

Oat (*Avena sativa* L.) is a recognized health-food, and the contributions of its different candidate A-genome progenitor species remain inconclusive. Here, we report chloroplast genome sequences of eleven *Avena* species, to examine the plastome evolutionary dynamics and analyze phylogenetic relationships between oat and its congeneric wild related species.

**Results:**

The chloroplast genomes of eleven *Avena* species (size range of 135,889–135,998 bp) share quadripartite structure, comprising of a large single copy (LSC; 80,014–80,132 bp), a small single copy (SSC; 12,575–12,679 bp) and a pair of inverted repeats (IRs; 21,603–21,614 bp). The plastomes contain 131 genes including 84 protein-coding genes, eight ribosomal RNAs and 39 transfer RNAs. The nucleotide sequence diversities (Pi values) range from 0.0036 (*rps19*) to 0.0093 (*rpl32*) for ten most polymorphic genes and from 0.0084 (*psbH-petB*) to 0.0240 (*petG-trnW-CCA*) for ten most polymorphic intergenic regions. Gene selective pressure analysis shows that all protein-coding genes have been under purifying selection. The adjacent position relationships between tandem repeats, insertions/deletions and single nucleotide polymorphisms support the evolutionary importance of tandem repeats in causing plastome mutations in *Avena*. Phylogenomic analyses, based on the complete plastome sequences and the LSC intermolecular recombination sequences, support the monophyly of *Avena* with two clades in the genus.

**Conclusions:**

Diversification of *Avena* plastomes is explained by the presence of highly diverse genes and intergenic regions, LSC intermolecular recombination, and the co-occurrence of tandem repeat and indels or single nucleotide polymorphisms. The study demonstrates that the A-genome diploid-polyploid lineage maintains two subclades derived from different maternal ancestors, with *A. longiglumis* as the first diverging species in clade I. These genome resources will be helpful in elucidating the chloroplast genome structure, understanding the evolutionary dynamics at genus *Avena* and family Poaceae levels, and are potentially useful to exploit plastome variation in making hybrids for plant breeding.

## Background

Most chloroplast (cp) genomes (plastomes) of land plants have a typical quadripartite structure with a pair of inverted repeats (IRs) separated by a large single-copy (LSC) region and a small single-copy (SSC) region, and genome size ranging from the reduced plastome of 85 kbp in the non-photosynthetic gymnosperm *Parasitaxus usta*, up to 218 kbp in *Pelargonium* [1, 2]. With high throughput sequencing methods [3], complete chloroplast genome sequences are widely used to improve phylogenetic resolution at the interspecific level and resolve the parentage of hybrid or polyploid taxa [4]. Some 866 plastid genomes of Poaceae have been assembled and deposited in the National Center for Biotechnology Information (NCBI:txid4479; accessed on 26 June 2020) Organelle Genome Resources since the publication of the first angiosperm chloroplast genome sequence of *Nicotiana tabacum* [5]. The massive complete chloroplast genome sequences, together with knowledge about chloroplast sequence variation, means complete plastid sequencing has become an effective tool for plant phylogenomic analysis.

The plastome datasets have been widely used for resolving the recalcitrant phylogenetic relationships in plants, in part because the plastome has long been considered as a single evolutionary unit, meaning that genes can be concatenated in order to dissect phylogenetic signals [6, 7]. Chloroplast genomes have also been identified with polymorphic regions caused by genomic expansions/contractions [8], inversions [9], gene rearrangements [10], etc. These mutational events in turn become synapomorphies for different plastid loci, sometimes yielding incongruent topologies. For example, three LSC inversions from *trnS-GCU* to *psbA* had been reported in some species of Poaceae [10], a LSC inversion from *trnE-UUC* to *rpoB* and a SSC inversion from *rps15* to *ndhF* were detected in some Asteraceae lineages [11, 12]. Whether such intermolecular recombinations exist in *Avena* plastomes remains uncertain, and their phylogenetic utilization merits further comparative study [13].

Oat (*Avena sativa* L., genomes AACCDD) is a recognized health food because of oat beta-glucan, which can actively reduce low-density lipoprotein cholesterol level and coronary heart disease risk [14]. The c. 29 species of *Avena* (Poaceae) are diverse in morphological and ecological terms, occurring across Asia, Europe and the Mediterranean Basin, Eastern Africa, and the Americas [15]. The genus includes diploids with A- or C-genomes (2*x* = 14), and a polyploid series with AB- or AC-genome tetraploids (4*x* = 28) and ACD-genome hexaploids (6*x* = 42) [16]. The A- and C-genome diploids are distinguished by the glume relative length and the chromosome structural differentiation [17], while the B and D genomes are not found in any extant diploids. The large size and highly repetitive nature of *A. sativa* genome has precluded the development of genomic breeding and selection of new oat varieties [18]. In recent phylogenetic analyses, oat was inferred to experience allopolyploidy events involving A-, C- and D-genome ancestors [16, 19], while the dynamic genomes with frequent chromosome translocations make it difficult to disentangle candidate progenitor species [20]. Plastid phylogenomics can be used for resolving contentious relationships [6, 7, 11], so the plastome-inferred phylogenies between oat and its wild relatives deserve detailed evaluation.

Comparative plastome studies show evolution of tandem repeats, insertions/deletions (indels) and single nucleotide polymorphisms (SNPs), and the role of tandem repeats in generation of substitutions and indels [21, 22], with certain plastome regions being predisposed to indel and substitution mutations. If the distribution of plastome repeat sequences can be determined, it is feasible to predict microstructural variations by the correlation analyses between repeats, indels and substitutions. In addition to the paucity of genomic resources, the A-genome lineage phylogeny is enigmatic in *Avena* [19, 23, 24]. Thus, it is important to fully address polymorphic regions of *Avena* chloroplasts in an evolutionary context.

In current study, we report the chloroplast genome structure characterization, the polymorphic regions, and plastid phylogenomics in *Avena* using new plastid sequences and comparisons with published sequences. Our objectives were to: (1) gain insight into plastome structure features; (2) examine the intermolecular combination and microstructural variation domains; and (3) understand the evolutionary dynamics in selected species among 25 published *Avena* plastomes.

## Results

### Plastome structure of *Avena* species

**Fig. 1:**
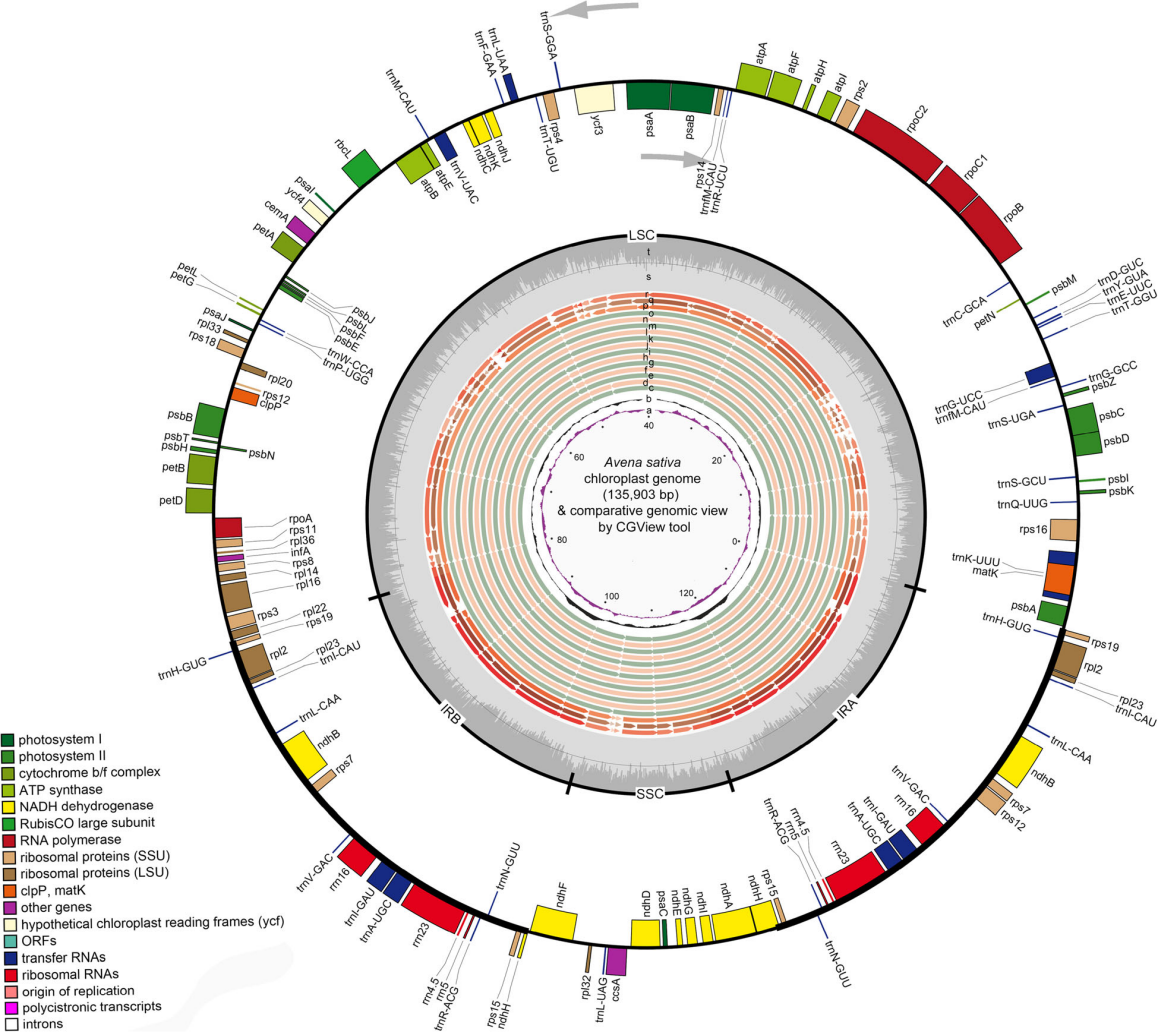
Chloroplast genome map of *Avena sativa*_Liu312 (GWHAOPK01000000; the outer circle and rings “s-t”) and GView comparison [25] of thirteen *Avena* species and three outgroup species plastomes (rings “c-r”). Genes belonging to different functional groups are shown in different colors. Genes shown on the outside of outer circle are transcribed counter-clockwise and on the inside clockwise. The tRNA genes are represented by one letter code of amino acids with anticodons. LSC, large single copy region; IR, inverted repeat; SSC, small single copy region. Rings “a-b” from the innermost ring denote GC skews and GC content deviations from *A. sativa*_Liu312 plastome GC content, respectively; rings “c-r” denote the plastome sequence comparison by BLAST between *A. sativa*_Liu312 and other species plastomes outwards in turn: *A. eriantha*_Liu435, *A. ventricosa*_Liu275, *A. atlantica*_Liu437, *A. wiestii*_Liu439, *A. strigosa*_Liu 315, *A. nuda*_Liu443, *A. hirtula*_Liu299, *A. sterilis*_NC031650.1, *A. sativa*_NC027468.1, *A. brevis*_Liu289, *A. sativa*_Liu312, *A. murphyi*_Liu442, *A. longiglumis*_Liu438, wheat (*Triticum aestivum*_NC002762.1), maize (*Zea mays*_NC00166.1), and rice (*Oryza sativa*_NC008155.1); plastome similar and highly divergent locations are represented by continuous and interrupted track lines (except for 14 non-overlapping tracking bins across 16 rings “c-r”), respectively, with a heuristic considering the total number of plastome bases (135,887 bp to 135,998 bp) contributing to hits and their scores with 0–14 non-overlapping 10 kbp tracking bins. Rings “s-t” denote AT and GC content of *Avena sativa*_Liu312 plastome (GWHAOPK01000000) by OGDRAW [26]

*Avena* plastomes display a typical quadripartite circular structure containing one large single copy (LSC), one small single copy (SSC), and two inverted repeat (IRB and IRA) regions by GView [25] and OGDRAW [26] (Fig. 1, Additional files 1, 2: Tables S1, S2). Eleven *Avena* plastome size ranges from 135,889 bp (*A. brevis*) to 135,998 bp (*A. wiestii*). Average GC content is 38.48% (Table 1). Average coverage depth ranges from 1634 × to 5339 × (Table 1, Additional file 12: Figure S1).

**Table 1:** The quantity and quality of the sequencing data and coverage depth of the assembled chloroplast genomes for eleven *Avena* species

Taxa	Voucher	Raw data (Gbp)	Clean data (bp)	Clean reads count	Chloroplast genome reads	Average coverage depth (×)	Maximum coverage depth (×)	Chloroplast genome size (bp)	GC content (%)	Genome Warehouse accession
*Avena atlantica*	Liu 437	32.6	26,768,534,000	107,074,136	2,903,043	5339	9373	135,940	38.48	GWHAOPC01000000
*A. brevis*	Liu 289	36.4	30,183,435,500	120,733,742	1,243,680	2288	2746	135,889	38.50	GWHAOPA01000000
*A. eriantha*	Liu 435	29.8	24,521,134,000	98,084,536	1,361,250	2504	5454	135,909	38.41	GWHAOPE01000000
*A. hirtula*	Liu 299	37.0	30,738,036,000	122,952,144	1,095,702	2015	2643	135,937	38.48	GWHAOPJ01000000
*A. longiglumis*	Liu 438	29.6	24,297,359,000	97,189,436	1,115,888	2052	4569	135,962	38.49	GWHAOPH01000000
*A. murphyi*	Liu 442	28.0	22,981,249,000	91,924,996	1,240,082	2281	5085	135,890	38.51	GWHAOPF01000000
*A. nuda*	Liu 443	41.9	33,688,961,000	134,755,844	1,773,953	3263	5956	135,935	38.48	GWHAOPD01000000
*A. sativa*	Liu 312	69.4	57,552,411,500	230,209,646	1,685,727	3101	4021	135,903	38.51	GWHAOPK01000000
*A. strigosa*	Liu 315	37.7	31,317,309,000	125,269,236	1,255,620	2309	2927	135,935	38.48	GWHAOPI01000000
*A. ventricosa*	Liu 275	17.4	14,267,461,000	57,069,844	1,039,219	1912	3178	135,910	38.41	GWHAOPG01000000
*A. wiestii*	Liu 439	15.4	12,685,761,500	50,743,046	888,788	1634	3004	135,998	38.48	GWHAOPB01000000

*Avena* plastomes contain the same set of 131 genes encoding 84 proteins, eight ribosomal RNAs (rRNAs) and 39 transfer RNAs (tRNAs) (Table 2). In *Avena* plastomes, small fragments of truncated *ndh* genes are detected in LSC (*ndhC*, 363 bp; *ndhJ*, 480 bp) and SSC regions (*ndhE*, 306 bp) while only partial *ndhH* in IRB region, the C (carboxy)-terminal part of *ndhH* is encoded by the SSC block and its N (amino)-terminal part by the neighbouring IRB region. The remaining *ndh* genes are the complete gene size.

**Table 2:** Genes present in *Avena* plastomes

Category of genes	Group of genes (gene number)	Gene name
Self replication	Ribosomal RNAs (8)	*rRNA16*^c^, *rRNA23*^c^, *rRNA4.5*^c^, *rRNA5*^c^
	Transfer RNAs (39)	*trnA-UGC*^a,c^, *trnC-GCA*, *trnD-GUC*, *trnE-UUC*, *trnF-GAA*, *trnfM-CAU*^e^, *trnG-GCC*, *trnG-UCC*^a^, *trnH-GUG*^c^, *trnI-CAU*^c^, *trnI-GAU*^a,c^, *trnK-UUU*^a^, *trnL-CAA*^c^, *trnL-UAA*^a^, *trnL-UAG*, *trnM-CAU*, *trnN-GUU*^c^, *trnP-UGG*, *trnQ-UUG*, *trnR-ACG*^c^, *trnR-UCU*, *trnS-GCU*, *trnS-GGA*, *trnS-UGA*, *trnT-GGU*, *trnT-UGU*, *trnV-GAC*^c^, *trnV-UAC*^a^, *trnW-CCA*, *trnY-GUA*
	Ribosomal protein (small subunit) (16)	*rps2*, *rps3*, *rps4*, *rps7*^c^, *rps8*, *rps11*, *rps12*^a,d^, *rps14*, *rps15*^c^, *rps16*^a^, *rps18*, *rps19*^c^
	Ribosomal protein (large subunit) (11)	*rpl2*^a,c^, *rpl14*, *rpl16*^a^, *rpl20*, *rpl22*, *rpl23*^c^, *rpl32*, *rpl33*, *rpl36*
	DNA dependent RNA polymerase (4)	*rpoA*, *rpoB*, *rpoC1*, *rpoC2*
	Translation-related gene (1)	*infA*
Genes for photosynthesis	Subunits of photosystem I (5)	*psaA*, *psaB*, *psaC*, *psaI*, *psaJ*
	Subunits of photosystem II (15)	*psbA*, *psbB*, *psbC*, *psbD*, *psbE*, *psbF*, *psbH*, *psbI*, *psbJ*, *psbK*, *psbL*, *psbM*, *psbN*, *psbT*, *psbZ*
	Subunits of cytochrome b/f complex (6)	*petA*, *petB*^a^, *petD*^a^, *petG*, *petL*, *petN*
	Subunits of ATP synthase (6)	*atpA*, *atpB*, *atpE*, *atpF*^a^, *atpH*, *atpI*
	Subunits of NADH dehydrogenase (13)	*ndhA*^a^, *ndhB*^a,c^, *ndhC*, *ndhD*, *ndhE*, *ndhF*, *ndhG*, *ndhH*^c^, *ndhI*, *ndhJ*, *ndhK*
	ATP-dependent protease subunit (1)	*clpP*
	Rubisco large subunit (1)	*rbcL*
Other genes	Maturase (1)	*matK*
	Envelop membrane protein (1)	*cemA*
	c-type cytochrome biogenesis (1)	*ccsA*
Genes of unknown function	Conserved open reading frames (2)	*ycf3*^b^, *ycf4*

^a^ Gene containing a single intron;

^b^ Gene containing two introns;

^c^ Two gene copies in the IRs;

^d^ Gene divided into two independent transcription units;

^e^ Duplicated gene in LSC region

In oat plastome, the *rpoC1* intron is absent (Additional file 13: Figure S2). The predicted amino acid sequence is extremely hydrophilic and anionic, suggesting that the corresponding region may be post-translationally removed. Neither sequences nor amino acids encoded by *rpoC1* gene have been altered excessively by the intron loss (Additional file 14: Figure S3). *ClpP* had lost its two introns in *Avena* plastomes. Nineteen genes are duplicated in IRs from *rps19* to *rps15*, including seven protein-coding genes (*rps19*, *rpl2*, *rpl23*, *ndhB*, *rps7*, *rps12*, and *rps15*), of which two use non-ATG start codons (*rps19*, GTG and *rpl2*, ACG; Table 2, Fig. 1). There are 16 different intron-containing genes, of which six are tRNA coding genes and *rps12* and *ycf3* contain two introns. The *trnK-UUU* has the largest intron (2435 bp) with *matK* located within it (Table 3).

**Table 3:** Genes with intron(s) in *Avena sativa* plastome

Gene	Region	Exon I (bp)	Intron I (bp)	Exon II (bp)	Intron II (bp)	Exon III (bp)
*atpF*	LSC	145^+^	825	407^+^		
*ndhA*	SSC	550^−^	1021	539^−^		
*ndhB*	IRB	777^−^	712	756^−^		
*ndhB*	IRA	777^+^	712	756^+^		
*petB*	LSC	6^+^	759	642^+^		
*petD*	LSC	8^+^	741	475^+^		
*rpl2*	IRB	391^−^	663	431^−^		
*rpl2*	IRA	391^+^	663	431^+^		
*rpl16*	LSC	9^−^	1052	402^−^		
*rps12*^a^	LSC + IRB	114^−^	–	232^−^	799	29^−^
*rps12*^b^	LSC + IRA	114^−^	–	232^+^	799	29^+^
*rps16*	LSC	40^−^	826	230^−^		
*trnA-UGC*	IRB	38^+^	811	35^+^		
*trnA-UGC*	IRA	38^−^	811	35^−^		
*trnG-UCC*	LSC	23^−^	677	48^−^		
*trnI-GAU*	IRB	37^−^	807	35^−^		
*trnI-GAU*	IRA	37^+^	807	35^+^		
*trnK-UUU*	LSC	37^−^	2435	35^−^		
*trnL-UAA*	LSC	35^+^	33	50^+^		
*trnV-UAC*	IRB	39^+^	596	37^+^		
*trnV-UAC*	IRA	39^−^	596	37^−^		
*ycf3*	LSC	126^−^	755	226^−^	725^−^	161^−^

Superscript^+^: exon is transcribed counter-clockwise in Fig. 1;

Superscript^−^: exon is transcribed clockwise in Fig. 1;

Hyphen–: spliceosomal intron;

^a,b^: The rps12 gene is divided into 5′-rps12 in LSC region, ^a^ 3′-rps12 in IRB region and ^b^ 3′-rps12 in IRA region

BLAST analyses of *Avena* species, wheat (*Triticum aestivum*_NC002762.1), maize (*Zea mays*_NC00166.1), and rice (*Oryza sativa*_NC008155.1) plastomes reflect the shared structural features. *Avena* plastomes share equivalent distribution patterns of GC islands where G and C are distributed unevenly between DNA strands (rings “a-b” in Fig. 1). The lower overall sequence identity is shared by wheat, maize, and rice (rings “p-r” in Fig. 1) compared to the congeneric *Avena* species. In *Avena*, a high level of similarity is restricted to IRs, and major differences originate from LSC and SSC regions. *Avena eriantha* and *A. ventricosa* plastomes share five highly divergent sequence locations in LSC region (*rps16-trnQ-UUG*, *trnC-GCA-rpoB*, *trnT-UGU-trnL-UAA*, *ycf4-cemA*, and *psaJ-trnP-UGG*) and one location in SSC region (*ndhF-rpl32*) to *A. sativa*_Liu312 plastome (rings “c-d” in Fig. 1). *Avena atlantica*, *A. wiestii*, *A. strigosa*, *A. nuda*, and *A. hirtula* share two highly divergent locations in LSC region (*rps16-trnQ-UUG* and *trnV-UAC-trnM-CAU*) and one highly divergent location in SSC region (*ndhF-rpl32*) to *A. sativa*_Liu312 plastome (rings “e-i” in Fig. 1). Wheat, maize, and rice outwards in turn according to the phylogenetic location.

*Avena sterilis*, *A. sativa*, *A. brevis*, *A. murphyi*, and *A. longiglumis* plastomes show high similarity in LSC, SSC and IR regions (rings “j-o” in Fig. 1), ribosomal gene clusters, tRNAs and the junction areas, where *rpl22*, *rps19*, *ndhH*, *ndhF*, and *psbA* are encoded (Additional file 15: Figure S4). Photosynthesis-related protein-coding genes in *Avena* plastomes show similarity to other angiosperms, including genes for ATP synthase, photosystem I and II, and RuBisCO. Other genes such as *rps3* and *ycf3* show less conservation.

### Evolution between monocot and dicot plastomes: *Avena* and *Taraxacum*

**Fig. 2:**
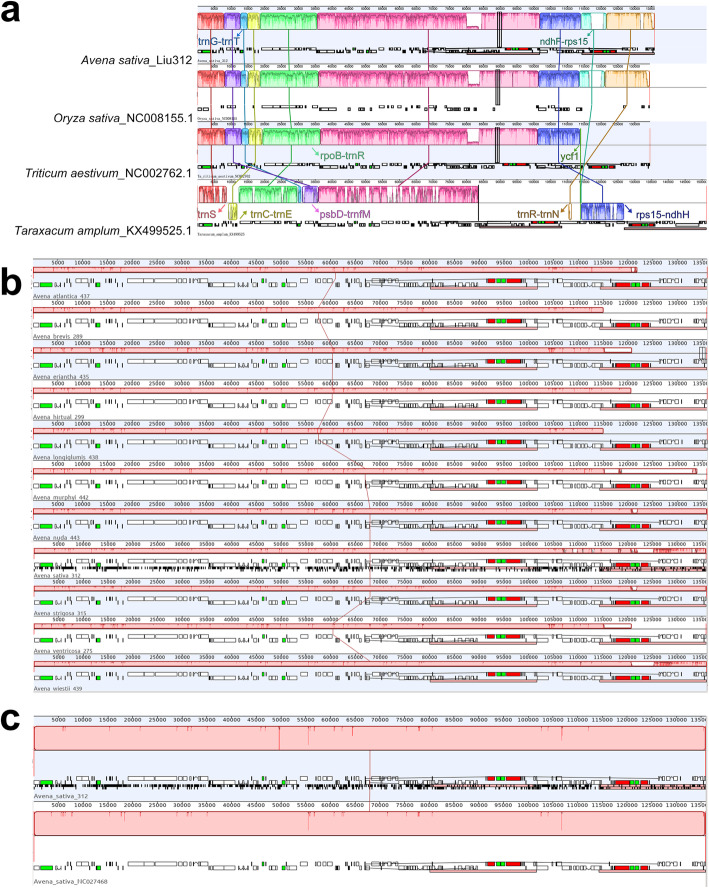
Mauve alignment. **a** Oat, rice, wheat, and dandelion (*Taraxacum amplum*) plastomes from this study and NCBI revealed similarities and differences in syntenical blocks. Two rearrangements with respect to the dicot plastome with LSC and IRB intermolecular recombination please see Fig. 3. **b** Mauve alignment of eleven *Avena* plastomes revealing no interspecific rearrangement. **c** Mauve alignment of *Avena sativa* (GWHAOPK01000000 and NC027468.1) plastomes revealing no intraspecific rearrangement. Each colored block is a region of collinear sequence among investigated species plastomes. Blocks shown above and below the line are in opposite orientations

**Fig. 3:**
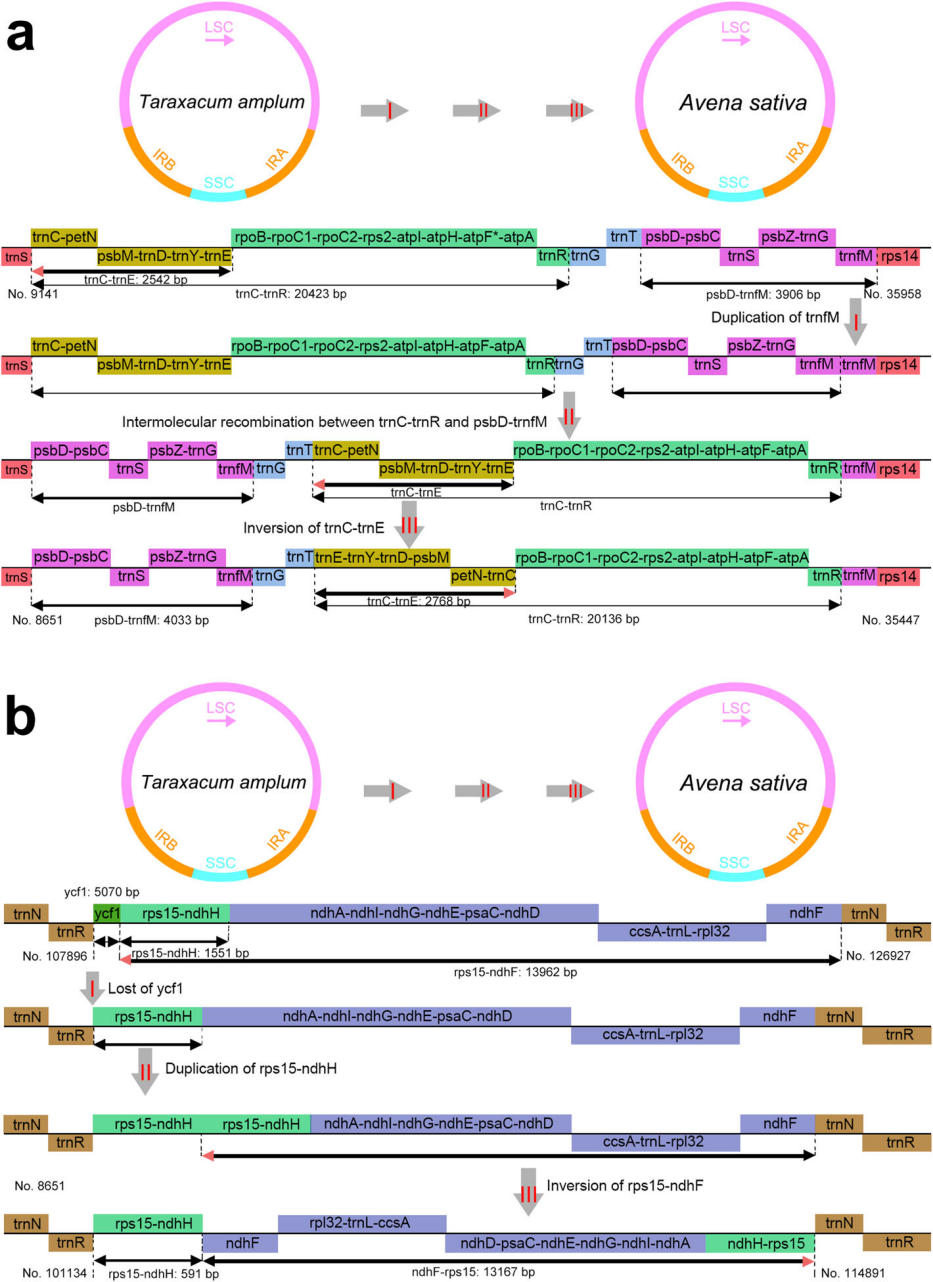
Schematic diagram showing postulated intermolecular recombination events across *Avena sativa* (top) and *Taraxacum amplum* (bottom). a Gene order and orientation within LSC intermolecular recombination. Roman numeral arrows denote the sequences of recombination events: I, Duplication of *trnfM* gene; II, Intermolecular recombination of *trnC-GCA-trnR-UCU* and *psbD-trnfM* regions; II, Inversion of *trnC-GCA-trnE-UUC* region. b Gene order and orientation within IRB intermolecular recombination. Roman numeral arrows denote the sequences of recombination events: I, Loss of *ycf1* gene; II, Duplication of *rps15-ndhH* region; III, Inversion of *rps15-ndhF* region. The vertical dashed lines indicate the rearrangement start-end base position. Gene transcribed in forward and reverse directions are indicated above and below the middle line, respectively

Mauve alignment [27] of plastomes show that the *Avena* plastome structure is similar to gramineous outgroups, *Cenchrus*, *Hordeum*, *Oryza*, *Saccharum*, *Secale*, *Sorghum*, *Triticum*, and *Zea*. At a higher resolution, two rearrangements are evident within LSC and IRB regions of oat, rice, wheat and *Taraxacum amplum* plastomes (Fig. 2a). There is no interspecific and intraspecific rearrangements within eleven *Aven*a species and two *A. sativa* accession plastomes (Fig. 2b, c). A scheme is shown where the monocot and dicot structures could be derived from each other by two intermolecular recombination events (Fig. 3): (1) LSC recombination region starting from a dandelion-like ancestral gene order with starting point from *trnC-GCA* to *trnfM* at one end, an initial duplication of *trnfM*, further recombination between *trnC-GCA-trnR-UCU* and *psbD-trnfM* (upstream), subsequently gave rise to the plastid DNA inversion of *trnC-GCA-trnE-UUC* occurred, encompassing about 31,415 bp sequence rearrangement of oat plastome (Fig. 3a); (2) IRB recombination region starting from a dandelion-like ancestral gene order with starting point from *ycf1* to *ndhF* at one end, an initial loss of *ycf1*, further duplication of *rps15-ndhH*, subsequently giving rise to the gene inversion of *rps15-ndhF* (downstream), encompassing about 13,758 bp sequence rearrangement of oat plastome (Fig. 3b).

**Fig. 4:**
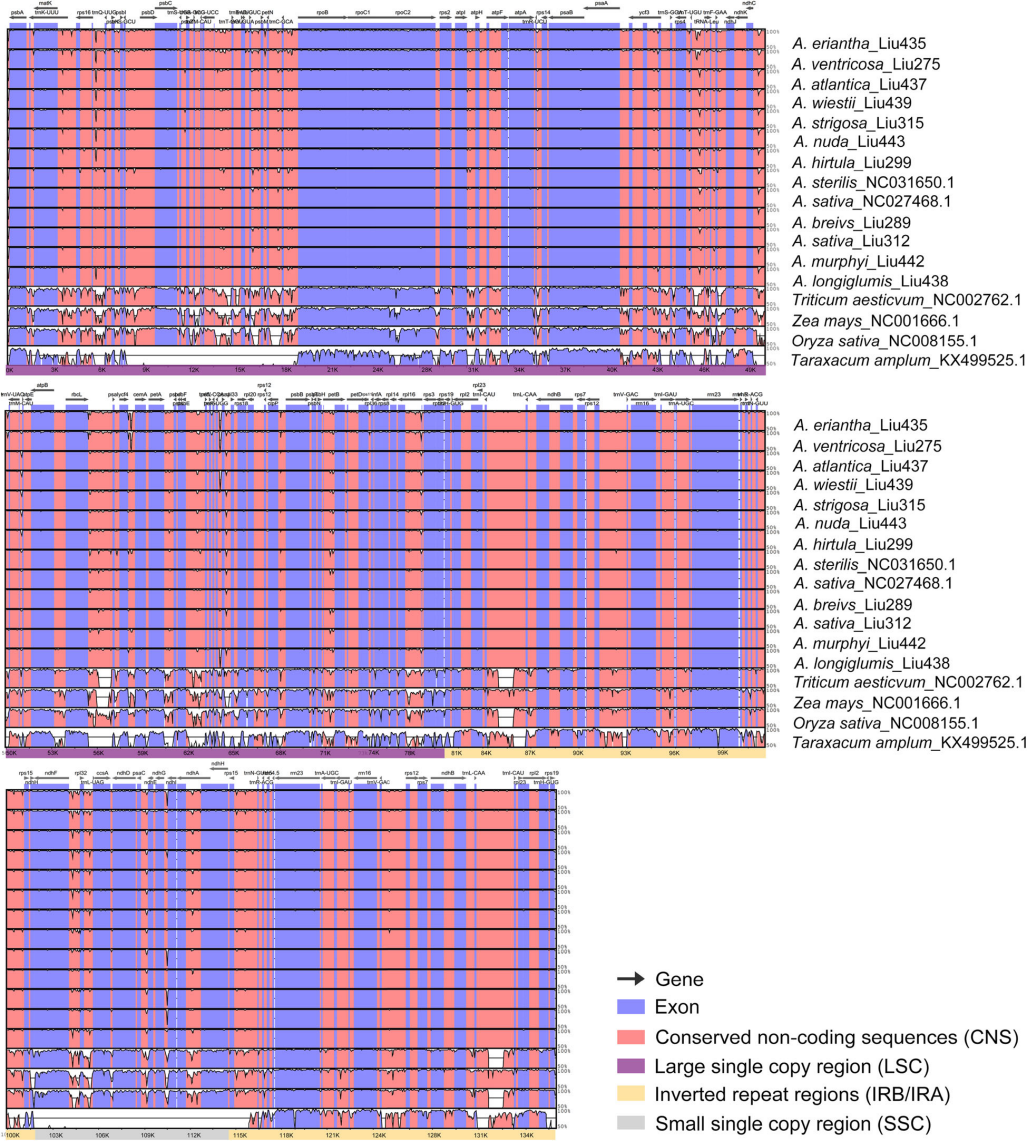
Sequence comparison of thirteen *Avena* species, wheat, maize, rice, and *Taraxacum amplum* plastomes. The mVISTA based similarity graphical information portrays sequence identity to *A. sativa*_Liu312 as a reference plastome. Grey arrows above the alignment denote the gene orientation. A cut-off of 50% identity is used for the plots. In each plot, the Y-scale axis represents percent identity (50 to 100%). Dashed rectangles indicate highly divergent regions compared with the reference plastome

**Fig. 5:**
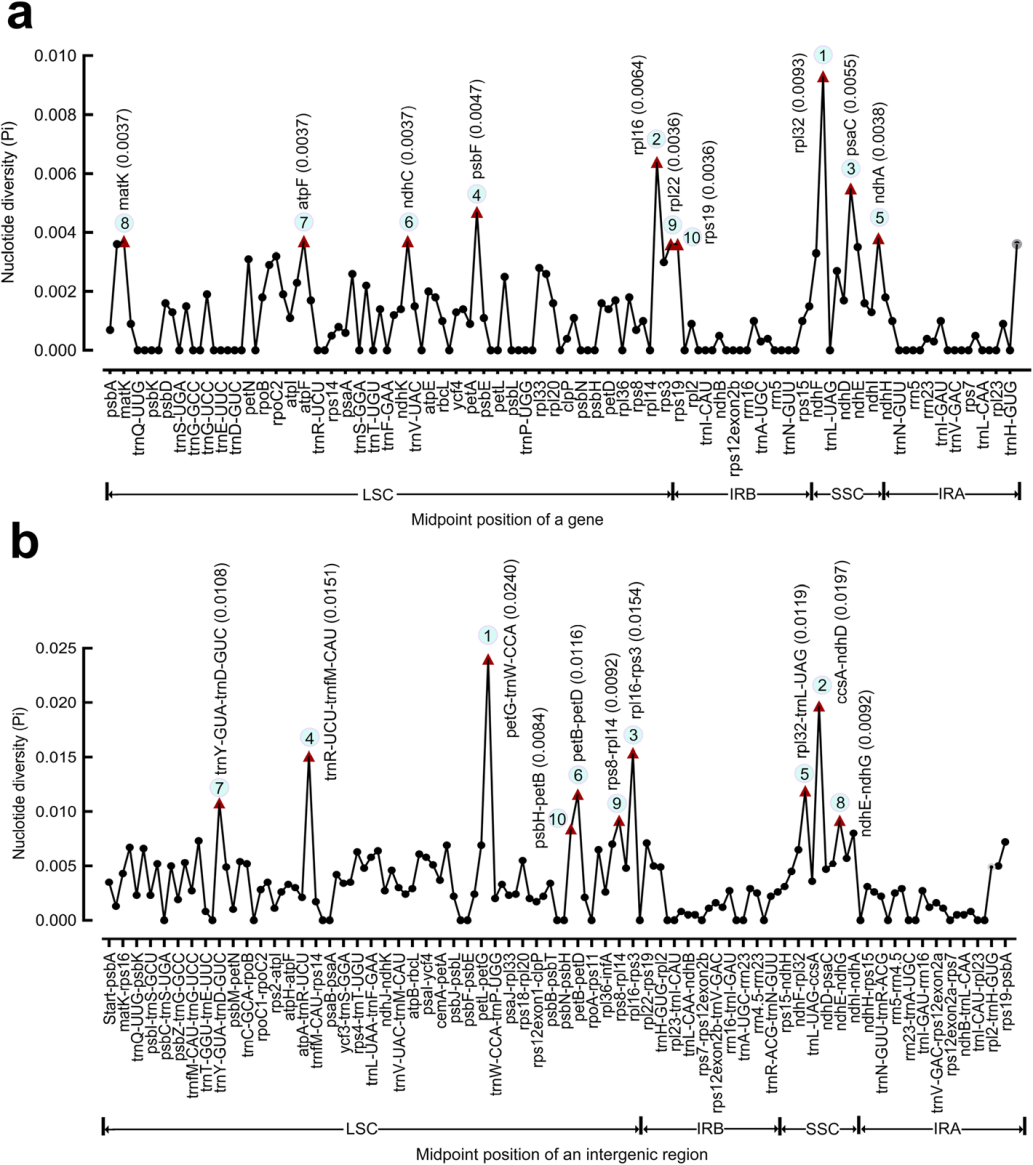
Nucleotide diversity (Pi) values using the aligned *Avena* plastome of (**a**) ten most polymorphic single copy genes and (**b**) ten most polymorphic intergenic regions. Regions are oriented according to the midpoint positions in plastome sequences with top 10 Pi values marked by red triangles

The mVISTA [28] analysis shows overall sequence identity and divergent regions in *Avena*. A high degree of synteny and gene order conservation indicate an evolutionary conservation at plastome level (Fig. 4). Notably, LSC and SSC regions are more divergent than IRs, and non-coding regions show a higher sequence divergence than in coding regions (Additional file 3: Table S3). DnaSP [29] analysis shows nucleotide diversity of single copy genes and intergenic regions. Ten most polymorphic genes in descending order include *rpl32*, *rpl16*, *psaC*, *psbF*, *ndhA*, *ndhC*, *atpF*, *matK*, *rpl22* and *rps19*, with the nucleotide diversity (Pi) values ranging from 0.0036 (*rps19*) to 0.0093 (*rpl32*) (Additional file 3: Table S3a, Fig. 5a). Among ten most polymorphic intergenic regions of *petG-trnW-CCA*, *ccsA-ndhD*, *rpl16-rps3*, *trnR-UCU-trnfM-CAU*, *rpl32-trnL-UAG*, *petB-petD*, *trnY-GUA-trnD-GUC*, *ndhE-ndhG*, *rps8-rpl14* and *psbH-petB*, with the nucleotide diversity (Pi) values ranging from 0.0084 (*psbH-petB*) to 0.0240 (*petG-trnW-CCA*) (Additional file 3: Table S3b, Fig. 5b). Six loci of LSC region and three loci of SSC region are identified for two datasets.

IR size is different among eleven *Avena* plastomes presented here available in Genome Warehouse database and fourteen species of different ploidies [representing a diploid maximum plastome size of 135,557 bp of *A. clauda* to a hexaploid maximum plastome size of 135,900 bp of *A. hybrida* among 25 *Avena* species] available in NCBI database (Additional file 15: Figure S4a, S4b), varying from 21,598 bp in *A. canariensis* to 21,619 bp in *A. clauda. Avena* plastomes have the same LSC/IRs and SSC/IRs borders, with two copies of *rps19* 36 bp from LSC/IRs borders, with LSC *rpl22* being 42 bp from LSC/IRB border, and LSC *psbA* being 86 bp from LSC/IRA border. For SSC/IRs, two copies of *ndhH* straddle SSC/IR borders, with 8 bp of upstream copy and 1001 bp of downstream copy moving into two sides of SSC region (except for 17 bp of upstream and 1022 bp of downstream copy in *A. hybrida*), and SSC *ndhF* being 69 bp from SSC/IRB border (except for 67 bp in *A. agardiana*) (Additional file 15: Figure S4b).

### Repeat sequence analysis

A total of 8234 (221 forward, 60 reverse, 192 palindromic and 7761 tandem) non-overlapped repeats were identified by using REPuter [30] and Phobos v.3.3.12 [31] for eleven *Avena* plastomes (Additional file 16: Figure S5a). Repeat numbers varied from 744 in *A. sativa* to 757 in diploids *A. eriantha* and *A. ventricosa*. Most abundant were tandem repeats (7–95 bp) ranging from 702 in *A. sativa* to 712 in *A. eriantha* and *A. ventricosa*, frequently with 7–10 bp nucleotides (Additional file 4: Table S4a, S4b computed by REPuter [30] and Phobos [31]). Reverse repeats were the least abundant repeats, ranging from 3 in *A. eriantha* and *A. ventricosa* to 11 in *A. nuda*, with 21–30 bp nucleotides (Additional file 4: Table S4b). For repeat distribution, 484 of 745 repeats (64.97%) were found in *A. hirtula* plastome LSC region, and 434 of 744 repeats (58.33%) were found in *A. sativa* plastome intergenic spacers (Additional file 4: Table S4c, Additional file 16: Figure S5b). For the tandem repeat distribution, 455 of 712 tandem repeats (64.63%) were found in LSC region of *A.ventricosa* plastome, and 417 of 703 tandem repeats (59.32%) were found in intergenic spacers of *A. brevis* plastome (Additional file 4: Table S4d, S4e, Additional file 16: Figure S5c).

There are 276 simple sequence repeats in *Avena* plastomes by Perlscript MicroSAtellite (MISA) [32], with the mononucleotide simple sequence repeats (SSRs) being the most abundant (Additional file 5: Table S5a). In each case, the 21–24 mononucleotide SSRs, one dinucleotide SSRs, and one-three composite SSRs are identified. No trinucleotide or tetranucleotide cpSSR exists in *Avena*. In general, the intergenic cpSSRs are more abundant than the genic cpSSRs in *Avena*, where the 15–20, four–five, and two–six SSRs are found in the intergenic, coding, and intron regions respectively (Additional file 5: Table S5b, Additional file 17: Figure S6). This also occurs in Asteraceae [11], Orchidaceae [33] and Fabaceae [34], probably due to an associated lower polymorphism of coding regions in contrast to non-coding regions. The C-genome diploids *A. eriantha* or *A. ventricosa* plastomes harbour 24 mononucleotide SSRs (92.31%) with 20 SSRs (77.00%) in the intergenic regions (Additional file 17: Figure S6). SSRs are more abundant in LSC region than in SSC and IR regions (Additional file 5: Table S5a). The majority of mononucleotide SSRs (17–20; 85–100%) are composed of A/T, which contributes to the base composition bias in *Avena* (A + T: 61.49–61.59%; Additional file 2: Table S2).

**Fig. 6:**
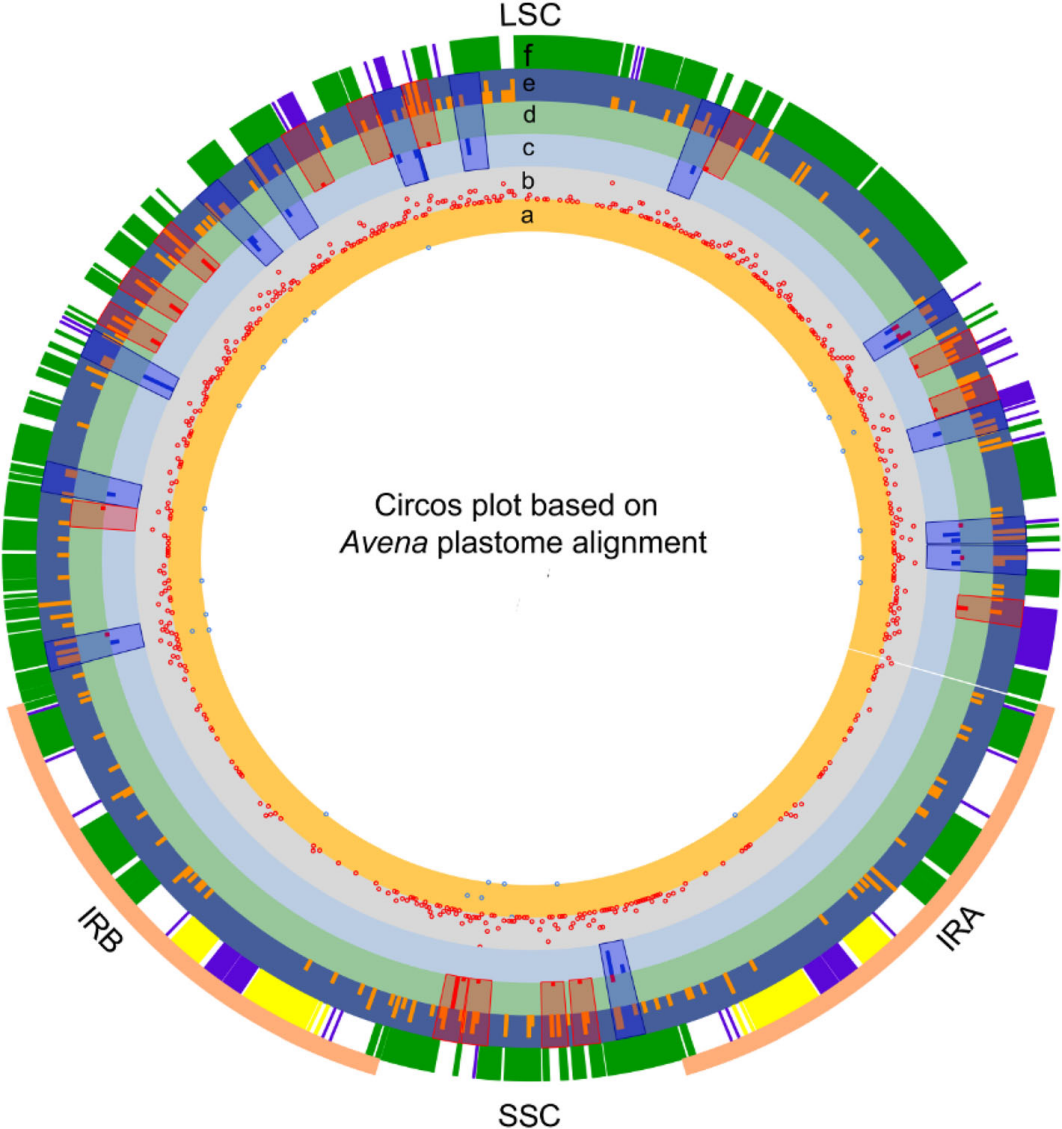
Circos plot based on complete chloroplast genome alignment between eleven *Avena* species presented here available in Genome Warehouse database and two *Avena* species available in NCBI (NC031650.1 and NC027468.1) with *A. sativa*_Liu312 as a reference. All data in rings showing the location relationship between tandem repeats, insertions/deletions (indels) and single nucleotide polymorphisms (SNPs) by R package circlize [35] in the non-overlapping 150 bp bins. For plastome alignment, rings “**a-b**” show the location of substitutions and SNPs, respectively. Dots with a high relative positions in rings “**a-b**” represent more polymorphic loci in the 150 bp window. Rings “**c-d**” show the location of deletions and insertions, respectively. Ring “**e**” shows tandem repeat (7 bp–95 bp) location by Phobos [31] with parameters of repeat length ≤ 100 bp and sequence identify ≥85%. The relatively height of columns in rings “c-e” represent the relative number of polymorphic loci belonging to deletions, insertions, or tandem repeats within the 150 bp window, respectively. Ring “f” shows *A. sativa*_Liu312 chloroplast map with coding genes labeled in green, rRNAs in yellow and tRNAs in blue. Blue shadows denote the surrounding location of tandem repeats and deletions; Red shadows denote the surrounding location of tandem repeats and insertions. LSC, SSC, IRs denote large single-copy, small single-copy, and inverted repeat regions of *Avena* plastome alignment. Total 702 tandem repeats, 141 insertions, 100 deletions, 992 SNPs, and 33 substitutions shown in the diagram

We have visualized the extent to which 868 tandem repeats and 221 indel mutations are nonnormally distributed among *Avena* plastomes within 150 bp windows (Additional file 7: Table S7) using R package circlize [35]. There are 81 insertions (66.94%) and 57 deletions (57.00%) located within tandem repeats (rings “c-e” in Fig. 6). We have explored the extent of genome-wide association between tandem repeats, indels and SNPs in *Avena* species alignments with *A. sativa*_Liu312 as a reference (Additional file 6: Table S6a, S6b). Due to the nonrandom distribution of mutation data in *Avena* plastomes, Spearman’s Rho correlations are performed among tandem repeats, indels and SNPs (Table 4). All these correlations are observed with high significance (*p* < 0.01). The average of correlations is stronger between tandems and indels, followed by tandems and SNPs then by indels and SNPs. The average value of correlations between tandems and indels is 0.3585, between tandems and SNPs is 0.2607, and between indels and SNPs is 0.2606 in thirteen *Avena* species. Mann-Whitney U test results show that a significant difference in the number of indels and SNPS from the tandem window and the non-tandem windows, with the corresponding *p*-vaules being 1.251 × 10^− 9^ and 1.477 × 10^− 9^, respectively.

**Table 4:** Spearman’s Rho correlation analysis result among tandem repeats, indels and SNPs using R v.3.5.3 [36] with correlation strengths of Akoglu [37] based on plastome alignments between eleven *Avena* species presented here available in Genome Warehouse database and two *Avena* species available in NCBI (NC031650.1 and NC027468.1) with *A. sativa*_Liu312 as a reference (150 bp windows)

	Tandem repeats and indels	Tandem repeats and SNPs	Indels and SNPs
Rho	0.3585	0.2607	0.2606
*p*-value	2.20 × 10^−16^***	1.48 × 10^−15^***	1.53 × 10^− 15^***

***Correlation was strongly significant at *p* < 0.01

### Gene selective pressure analysis

All protein-coding genes went through purifying selection: only 27 coding genes have non-synonymous/synonymous mutation (Ka/Ks) ratio < 1, with Ka/Ks ratio ranging from 0.0313 (*rps3*) to 0.8896 (*rpoC2*) (Additional file 8: Table S8). The *rps3* has no nonsynonymous rate change, and *atpE*, *atpI*, *ndhD*, *ndhH*-SSC, *rbcL*, *rpl32*, *rpl33*, *rpoA*, *rps11*, *rps2*, and *ycf4* have no synonymous and non-synonymous changes (Additional file 18: Figure S7). Ka/Ks ratios of photosynthesis loci including *psbA* (0.1354), *petA* (0.1416–0.2789), two subunits of ATP synthase genes (*atpE* and *atpI*) range from 0.1585 to 0.1632. Ka/Ks ratios of four subunits of *ndh* genes (*ndhD*, *ndhF*, *ndhH*-SSC and *ndhI*) range from 0.0353 to 0.2351. Ka/Ks ratios of self-replicating genes are as follows: 0.0313–0.2763 of ribosomal protein small subunit genes (*rps2*, *rps3*, and *rps11*), 0.2405–0.2621 of ribosomal protein large subunit genes (*rpl32* and *rpl33*); 0.0446–0.8896 of DNA dependent RNA polymerase genes (*rpoA*, *rpoB*, *rpoC1* and *rpoC2*); and 0.1395–0.2840 of *infA*. Ka/Ks ratios of other genes are 0.2548–0.7669 of *matK*, 0.1334–0.1775 of *ccsA* and 0.1550 of *ycf4*. The *atpF*, *matK* and *rpoC2* genes in LSC region have a faster divergence than those genes in SSC or IRB regions. The *rpoC2* gene is the fastest evolving gene in A-genome diploids *A. brevis* and *A. longiglumis* (Additional file 18: Figure S7). Coding genes in IRs (*rps19*, *rpl2*, *rpl23*, *ndhB*, *rps7*, *rps12* and *rps15*) do not show Ka and/or Ks rate changes.

### Codon usage bias

**Fig. 7:**
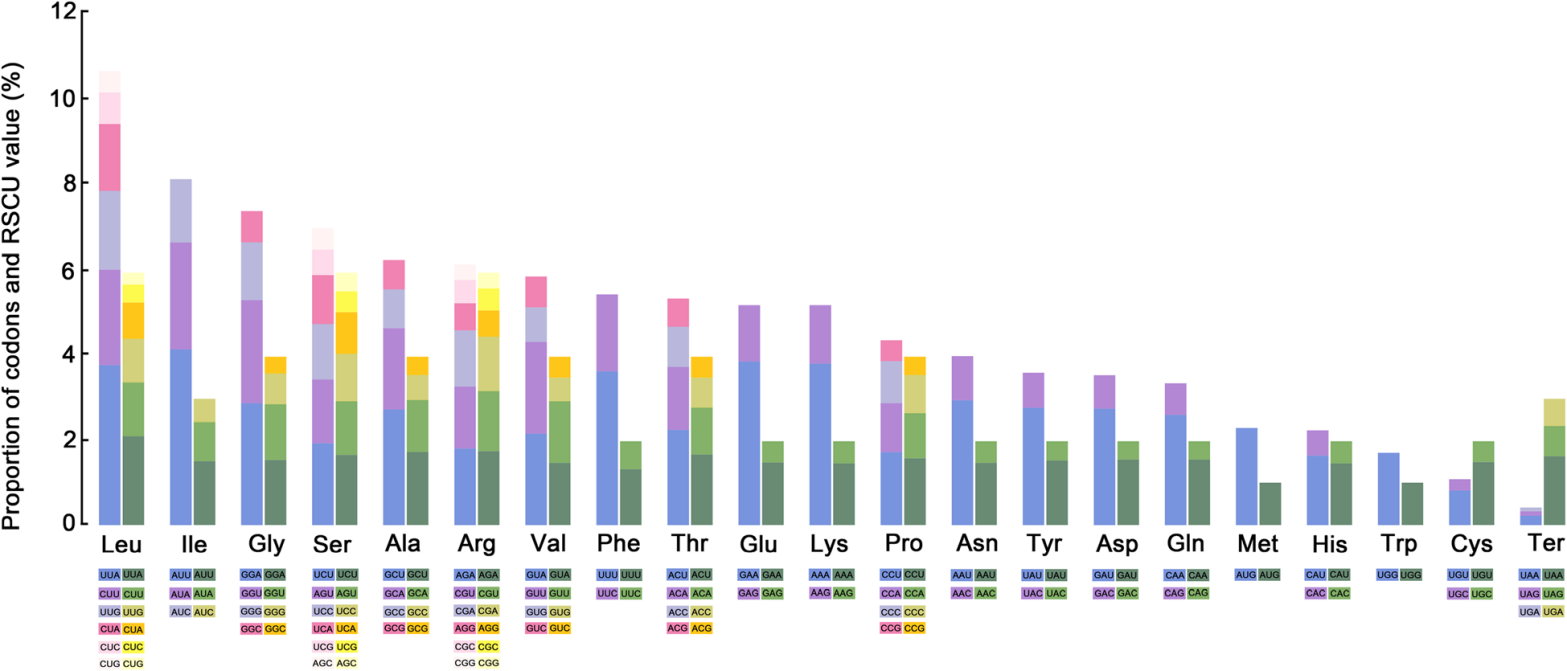
Codon content of 20 amino acids and stop codons in protein-coding genes of *Avena* plastomes. The histogram on the left-hand side of each amino acid denotes codon usage within *Avena* plastomes, and the right-hand side denotes the codon RSCU [38] values. Colors correspond to codons listed underneath the columns

The protein-coding genes present a total of 19,913 codons in *A. brevis* plastome to 19,921 in *A. eriantha* and *A. ventricosa* plastomes (Additional file 8: Table S8a) with the RSCU (relative synonymous codon usage) values [38] ranging from 0.28 (CUG) to 2.12 (UUA) (Additional files 8, 9: Tables S8b, S9a, S9b, Fig. 7, Additional file 19: Figure S8). Leucine (10.76–10.78% of each species) and cysteine (1.09% of each species) are the most and the least abundant amino acids except for stop codons (0.42% of each species; Additional file 9: Table S9b). Our findings match the trend reported across other angiosperm plastomes [7], which show leucine and isoleucine to be the most common codons. No codon bias can be shown by methionine (AUG) and tryptophan (UGG), encoded by only one codon. Codon usage is biased towards A and T at the third codon position (Additional file 10: Table S10a, S10b, Additional file 19: Figure S8). The AT content for first, second and third codons average 52.7, 60.3 and 70.0% in *Avena* (Additional file 8: Table S8a), respectively. Almost all A/U-ending codons have RSCU values larger than one except for *trnL-UAG* and *trnS-UGA* (RSCU = 0.87, 0.99 respectively), whereas all of C/G-ending codons have RSCU values ≤1 in *Avena* species (Additional file 11: Table S11a, S11b).

### Phylogenomic *a*nalyses

**Fig. 8:**
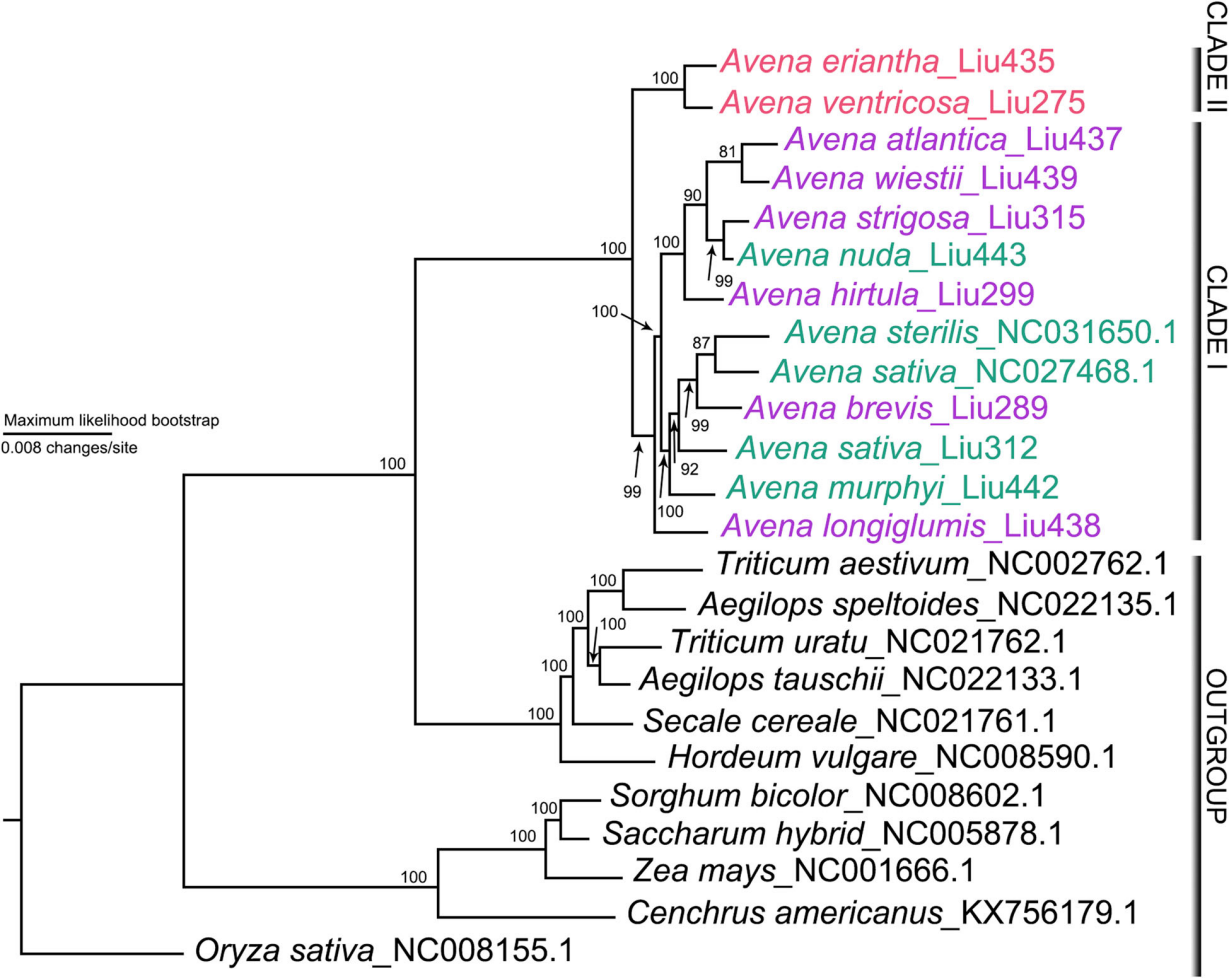
Maximum likelihood tree inferred from *Avena* plastome sequences. Two clades are identified: clade I includes A-genome diploids, tetraploid *A. murphyi* and hexaploid *A. sativa*, and clade II includes C-genome diploids. Node support denotes the maximum likelihood bootstrap value. Pink, red and green taxa correspond to A-, C-genome diploid and polyploid species in *Avena*, respectively

Our phylogenomic analyses substantially increased resolution and provided robust phylogenetic relationships in *Avena* (Fig. 8). The monophyly of *Avena* received strong maximum likelihood bootstrap support (MLBS = 100%). Two strongly supported infrageneric lineages (MLBS = 100%) are identified in *Avena*: clade I contains the A-genome diploid-polyploid subclade I (*A. sativa*, *A. sterilis*, *A. murphyi*, and *A. brevis*), subclade II (*A. nuda*, *A. atlantica*, *A. hirtula*, *A. strigosa*, and *A. wiestii*), and the basal position of *A. longiglumis* received strong support (MLBS = 100%); and clade II contains the C-genome diploid lineage (*A. eriantha* and *A. ventricosa*) (Fig. 8).

Maximum likelihood (ML) topologies were constructed using not only the combined sequences of LSC and IRB intermolecular recombination fragments (28,164 bp, 14,262 bp), but also those of combined ten most polymorphic genes (11,028 bp, 8212 bp) and those of combined ten most polymorphic intergenic regions (3099 bp, 2374 bp) in order to develop suitable plastid markers for inferring the interspecific phylogenetic relationships (Additional file 20: Figure S9 [39–41]). Numbers above nodes are the trustable Shimodaira-Hasegawa test-approximate likelihood-ratio test support/Ultrafast bootstrap support (SH-aLRT > 80.0%, UFBoot > 80.0%). Phylogenetic tree of LSC intermolecular recombination fragments is consistent with complete plastome ML tree topology (Additional file 20: Figure S9a), showing the basal position of *A. longiglumis* within clade I with strong support (SH-aLRT = 100%, UFBoot = 100%). The remaining topologies show that *A. longliglumis* is sister to subclade I with medium support (SH-aLRT = 82.8%, UFBoot = 86.0%) by IRB intermolecular recombination fragments (Additional file 20: Figure S8b) with weak support (SH-aLRT < 50.0%, UFBoot < 50.0%) and by the phylogenetic topologies of protein-coding genes and intergenic regions with high nucleotide diversities (Additional file 20: Figure S9b-S9f). ML topology is further constructed using plastomes of *Avena* species presented here available in Genome Warehouse and 25 *Avena* species available in NCBI [19, 24]. It is without significant topological change compared to a previous Bayesian maximum clade credibility (MCC) tree [19]. Namely, clade I contains the *A. sativa* inserted subclade I (from *A. agadirianan* to *A. sterilis*), the *A. nuda* inserted subclade II (from *A. atlantica* to *A. nuda*) and the basal position of *A. longiglumis* together with extra three diploid species (*A. canariensis*, *A. damascene* and *A. lusitanica*) in ML tree of 38 *Avena* plastomes (Additional file 21: Figure S10).

## Discussion

### Plastome evolution

All *Avena* plastomes possessed the typical gramineous plastome structure including the LSC and IRB intermolecular recombinations that are present in nearly all Poaceae, e.g. *Oryza*, *Hordeum*, *Triticum*, *Secale*, *Cenchrus*, *Saccharum*, *Sorghum* and *Zea* (Fig. 3). Liu et al. [11] suggested that the LSC recombination region has the gene (partially) missing in certain lineages, e.g. the missing *clpP* introns or *rpoC1* intron, and this could be a particularly active region for sequence rearrangement in Poaceae plastomes [42]. *Avena* plastome average GC content (38.48%) is similar to other monocot plastomes, such as *Elodea canadensis* (37%) [43], *Smilax china* (37.25%) [11], and *Najas flexilis* (38.2%) [44]. According to report, 70 up to 88 protein-coding genes are present in angiosperm plastomes [45], there are 84 such genes in *Avena* plastomes. The *ndh* gene size and content vary widely among some heterotrophic species, which retain photosynthetic ability due to the non-functional role of *ndh* genes [44]. In the Asteraceous dandelion (*Taraxacum amplum* Markl. in *T. officinale* F.H. Wigg. agg.) plastome, *rpoC1* is interrupted by a ∼ 680 bp intron, and two exons expand for 450 and 1638 nt respectively [12]. In oat plastome, the *rpoC1* intron is absent. Intron excision from the primary transcript by splicing represents a prerequisite for translation of messenger RNAs (mRNAs) into the correct full-length protein, while the selective pressure of group II intron splicing events may have been important and their role underappreciated [46]. The *rps12* is trans-spliced with exon 1 coded in LSC region and exons 2 and 3 in IRs. Spliceosomal introns are a feature of eukaryotic nuclear genes, and intron splicing can enhance gene expression [47]. Other aspects of mRNA metabolism, including pre-mRNA polyadenylation, editing transcription and mRNA decay are also influenced by removal of introns by the spliceosome [48].

The LSC/IRs and SSC/IRs borders are relatively conserved among angiosperm plastomes, mostly positioned within *rps19* or *ycf1* [49]. Compared with the ancestral angiosperm genome structure (represented by *Nicotiana tabacum* [50]), the *Avena* plastome IRs have expanded into the LSC region, resulting in the movement of *rps19*. Significant expansions have been reported in other plants, such as the extreme 50 kbp expansion found in *Pelargonium* × *hortorum* L.H. Bailey [51], and the 4 kbp expansion in *Jasminum nudiflorum* Lindl [52].. Here, no significant IR length variation was detected among *Avena* plastomes.

It is well known that certain plastome regions show different mutation rates. Dispersed repeats may facilitate intermolecular recombination and plastome diversity creation, because the genome regions with increased sequence diversity could be formed by repeat sequence abundance in prokarya and eukarya [53]. The cpSSRs and indels are mainly distributed in the non-coding regions of *Avena* plastomes, the similar distribution preference of cpSSRs and indels has been reported in *Olea europaea*, *Pseudoroegneria libanotica* and *Salvia miltiorrhiza* [54–56].

The nonrandom distribution of tandem and mutation locations have been found in *Avena* plastome visualization (Fig. 6), and correlations at the interspecific level also exhibits variations. We observe moderate correlations between tandems and indels, and weak correlations between tandems and SNPs and between indels and SNPs in *Avena* plastomes. Comparisons of tandem-presence-bin and tandem-absence-bin show strong difference for numbers of indels and SNPs, these differences are also statistically significant (*p* < 0.001) by Mann-Whitney U test results. Araceae and Malvaceae plastomes have associations between repeats, indels and substitutions [21, 22], and the adjacent position relationships suggest a role for tandem repeats in SNP and indel mutations in *Avena*. These results support the hypothesis that tandem repeats play an important role in causing plastome mutations [21].

All protein-coding genes were found to be under purifying selection. This pattern has also been demonstrated in other Poaceae plastomes such as rice, maize and wheat [57], reflecting the typically conservative plastid genome across most angiosperms. Changes of nucleotide substitution rates have been correlated with obligate parasitism rather than loss of photosynthesis [58]. In parasites, there is less selective pressure on plastid function, so purifying selection on genes encoding proteins for DNA maintenance and expression may be relaxed. Increasing specialization on external carbon (e.g., improved nutrient acquisition efficiency) thus leads to changes of evolutionary rates of plastid housekeeping machinery because of the lifestyle, although with most plastid proteins being encoded in nucleus, the selection strength at protein-coding loci in plastids is unclear.

### Phylogenetic analysis in *Avena*

To resolve relationships among closely related diploid species, it is imperative to identify rapidly evolving loci [11]. Concordant interspecific relationships are recovered by the phylogenies inferred from LSC intermolecular recombination fragment and from complete plastome sequences. There might be evolutionary convergence between the fragment from *psbD* to *trnR-UCU* and complete plastome sequences as a result of gene interaction and co-evolution to conserve the chloroplast functions. The complete plastome ML tree supports the basal position of *A. longiglumis* within clade I with strong support (MLBS = 100%; Fig. 8), and those highly supported within the A-genome diploid-polyploid lineage in previous studies [19, 24] and within clade I of ML tree of 38 *Avena* plastomes (Additional file 21: Figure S10). Diploid *A. longiglumis* together with *A. canariensis*, *A. damascena* and *A. lusitanica* are proposed to be oat potential ancestors [19, 24], whose contributions for oat speciation are deserve further investigation in the context of genomics and cytogenetic data.

Plastome marker selection should be made based on appropriate evolutionary rates (Pi values) are appropriate. For subclade I, *A. sativa* is sister to diploid *A. brevis* and tetraploid *A. murphyi* based on the ten most polymorphic coding regions and non-coding regions respectively (Additional file 20: Figure S8c, S8d). Such variation indicates that *A. brevis* and *A. murphyi* might carry a diverged A-genome from the most likely A-genome diploid ancestor, *A. longiglumis*, supported by nuclear gene *Pgk1* too [23]. It is also evident that the most frequently used chloroplast markers (including *trnL-trnF* and *matK*) show few polymorphisms (0.0058, 0.0037) at the interspecific level with respect to adding outgroup wheat (0.0166, 0.0136). The nucleotide diversity for 30 most polymorphic intergenic regions ranged from 0.0287 to 0.1100 in *Hibiscus* (Malvaceae) [59], substantially higher than in *Avena* top 30 intergenic regions with nucleotide diversity ranging from 0.0053 to 0.0240. Eleven loci, including *petG-trnW-CCA*, *ccsA-ndhD*, *rpl32-trnL-UAG*, *trnY-GUA-trnD-GUC*, *rps16-trnQ-UUG*, *infA-rps8*, *petA-psbJ*, *trnF-GAA-ndhJ*, *rps4-trnT-UGU*, *ndhG-ndhI* and *ndhF-rpl32*, are shared by the two genera. Among the 10 loci, *rpl32-trnL-UAG* is also shared by *Fritillaria* [60], and *rps16-trnQ-UUG* is also shared by *Artemisia* and *Dendrobium* [61, 62]. In addition, *ccsA-ndhD*, *ndhF-rpl32*, *psbK-psbI* are shared by *Avena* and *Artemisia* [61], and *petN-trnC-GCA*, *rps12-clpP*, and *petL-petG* are shared by *Avena* and Monsteroid species [63]. These markers could be used for the deep divergence in the family level as mutational hotspots but not for *Avena*.

## Conclusion

Diversification of *Avena* plastomes is explained by the presence of highly diverse genes and intergenic regions, LSC intermolecular recombination, and the co-occurrence of tandem repeats and indels or single nucleotide polymorphisms. The study demonstrates that the A-genome diploid-polyploid lineage maintains two subclades derived from maternal ancestors, and the diverged A-genomes originate from the diploid *A. longiglumis*, the optimum candidate ancestor for the A-genome in polyploid species. The genus does deserve attention as a model system to understand the underlying mechanisms of plastome evolution, because of the need to mine genetic resources from both chloroplast and nuclear genomes for crop variety breeding.

## Methods

### Isolation of DNA and sequencing

Seeds of eleven *Avena* species (sample origin and genome designation [16] given in Additional file 1: Table S1) were obtained from CN-Saskatchewan and USDA-Beltsville Germplasm System in this study. Healthy leaf samples were collected from South China Botanical Garden Greenhouse in Guangzhou, China. Whole genomic DNA was extracted from 100 mg fresh leaf tissue using the DNeasy Plant Mini Kit following the manufacturer’s protocol (Biomed, Beijing, China).

DNA libraries were prepared and sequenced with the Illumina HiSeq2500 platform (Illumina, San Diego, CA, USA) for *A. brevis*, *A. hirtula*, *A. strigosa* and *A. sativa* with PE250 bp reads from 300 bp insert libraries and the remaining seven species with PE250 bp reads from 500 bp insert libraries. Project data have been deposited at the Genome Sequence Archive (GSA) of National Genomics Data Center with accession number CRA003107 (https://bigd.big.ac.cn/search/?dbId=&q=CRA003107). Three coding gene sequences with the highest coverage were used the seed sequence for de novo assembly of *Avena* plastome by NOVOPlasty v.2.6.2 [64] with *A. sativa* (NC027468.1) as a reference for IR regions correction (Fig. 1). Then Geneious R11 [65] was used for contigs merging and de-redundancy. Chloroplast circularization and initiation site determination were manually processed.

### Plastome assembly and annotation

Many protocols are available for chloroplast genome assembly including SOAPdenovo2 [66] and Velvet [67]. Velvet is suitable for small genome assembly, and we found SOAPdenovo2 and Velvet cannot independently assemble one complete plastome sequences of *Avena* without auxiliary gap-filling softwares. Since NOVOPlasty v.2.6.2 [64] is capable of extending one read into a complete circular genome through extending the given seed to form circular genome, three single copy coding gene sequences with the highest coverage were used as starting seed sequences for de novo assemblies of *Avena* plastomes by NOVOPlasty separately with k-mer values of 79, 89, 99 and 109 (Fig. 1). Chloroplast cyclization and initiation site determination were optimized manually. The integrity of *Avena* plastomes was also verified by Velvet contigs covered 99% of the NOVOPlasty assemblied plastomes. Average coverage depth (1634 × to 5339 ×) was calculated by Geneious R11 [65] by mapping the total clean reads to de novo assembled plastome of each species (Table 1, Additional file 12: Figure S1).

The chloroplast genes were annotated by GeSeq [68] with default parameters to predict protein-coding genes, rRNA and tRNA genes (Tables 2, 3, Additional file 13: Figures S2). IRs were confirmed by IR finder [69]. All tRNA genes were further verified by using tRNAscan-SE (http://lowelab.ucsc.edu/tRNAscan-SE/). GC content was calculated by Geneious R11 [65]. The circular chloroplast genome map was drawn by OrganellarGenomeDRAW (OGDRAW) v.1.3.1 [26] followed manual optimization (Fig. 1). Plastomes were submitted to Genome Warehouse in National Genomics Data Center (Additional file 1: Table S1).

### Complete chloroplast genome comparison

The data gave maximum coverage depth of 2643 × to 9373 × for *Avena* plastomes (Additional file 12: Figure S1). Chloroplast genome similarity of eleven *Avena* species, wheat, maize, and rice were assessed using BLASTN by GView with 10 kbp connection windows [25]. The plastomes of eleven *Avena* species, two accessions of *A. sativa*, gramineous outgroups and *Taraxacum amplum* were respectively aligned by Mauve [27] in order to investigate intermolecular recombination events (Figs. 2, 3).

Plastome structures among *Avena* species, wheat, maize, rice, and dandelion were compared by mVISTA percent identity plot in Shuffle-LAGAN mode [28, 70] to reveal several major genomic variations located in LSC and SSC regions (Fig. 4). Subsequently, nucleotide diversity of single copy genes and intergenic regions was estimated for two sequence datasets (thirteen *Avena* species+*Triticum aestivum* plastomes) by DnaSP v.6 [29] (Additional file 4: Table S4, Fig. 5). Plastome genetic architecture of eleven *Avena* species available in Genome Warehouse, *Triticum aestivum* plastomes and fourteen *Avena* species available in NCBI for LSC/IRs and SSC/IRs borders were analysed by IRscope [71] (Additional file 15: Figure S4).

### Repeat structure identification

Plastome repeat sequences were identified by REPuter [30] (repeat unit length minimum ≥21 bp, repeat identity ≥90%, Hamming distance 2). Four matches of repeats were classified as follows: (i) forward, (ii) reverse, (iii) complement, and (iv) palindromic match (Additional file 4: Table S4a, S4b, S4c, Additional file 16: Figure S5a, S5b, S5c). Tandem repeats (13–75 bp) were identified by using Phobos v.3.3.12 [31] (Additional file 4: Table S4d, S4e). Simple sequence repeats (SSRs) were examined by Perlscript MicroSAtellite (MISA) [32] with parameters setting as follows. The motif sizes were one to six nucleotides, and the minimum repeat unit was defined as 10 for mononucleotides, six for dinucleotides, and five for tri-, tetra-, penta-, and hexa-nucleotides (Additional file 5: Table S5a, S5b, Additional file 17: Figures S6). Repeat type and distribution were analysed by GraphPad Prism v.8.0. Selecting *A. sativa*_Liu312 plastome as a reference for coordinate positions, tandem repeats were detected by Phobos [31], and indels and SNPs were counted within the non-overlapping 150 bp window for thirteen *Avena* plastomes using Mauve pairwise alignment (Additional file 6: Table S6a, S6b).

Circos plot, showing the locations and relationships between tandem repeats, insertions/deletions (indels), and single nucleotide polymorphisms (SNPs), was generated by R package circlize [35]. Comparative plastome studies show that certain plastome regions are predisposed to mutations due to nonrandom distribution of tandem and mutation locations (Fig. 6), so Spearman’s Rho correlation analyses are performed to measure the degree of association between tandems and mutations and between mutations by R v.3.5.3 [36]. The strengths of Spearman rank correlations between two variables have set threshold values as follows: negligible or very weak (0.1–0.19), weak (0.20–0.29), moderate (0.30–0.39) and strong (0.4–0.69) [37]. The probability (*p*) of significance of the correlations was tested at *α*-level of 0.01. Mann-Whitney U tests are further performed to decide whether tandem repeat presence/absence affects the numbers of indels and SNPs in each 150 window by R v.3.5.3 (Additional file 7: Table S7), using grouping variables (tandem repeat number > 0 or = 0) and outcome variables (indel number and SNP number) in each 150 bp window.

### Synonymous codon usage bias

To investigate the selective pressure on plastome protein-coding genes between two species, Ka/Ks values were calculated by KaKs_Calculator 2.0 [72] (Additional file 8: Table S8a, Additional file 18: Figure S7). RSCU values were examined by CodonW v.1.4.2 [38]. Codon absolute number and codon usage patterns were analysed by GraphPad Prism v.8.0 (Additional files 9, 10, 11: Tables S9, S10, S11; Fig. 7, Additional file 19: Figure S8).

### Phylogenomic analysis

Phylogenetic trees were constructed by ML analyses using *Avena* and gramineous outgroup plastome sequences (162,505 bp) based on recent phylogeny [73]. The sequences were aligned and then manually adjusted by BioEdit [74]. The best-fitting nucleotide substitution model was determined using the Akaike Information Criterion (AIC) in iModeltest v.2.1.10 [75]. The GTR + I + G model was used in ML analyses, which were performed by MEGA v.6.0 with 1000 bootstrap replicates [76].

In order to evaluate the alternative hypotheses of phylogeny, ML trees were constructed using not only the combined sequences of LSC or SSC intermolecular rearrangement fragments (28,164 bp, 14,262 bp), but also those of combined ten most polymorphic protein-coding genes (11,028 bp, 8212 bp) and combined ten most polymorphic intergenic regions (3099 bp, 2374 bp) (Table S4, Fig. 8, S8) by IQ-TREE v.1.6.8 [39] employing the model-testing function to infer the best-fit substitution model for such six datasets under the Bayesian information criterion. To evaluate node reliability, we implemented a Shimodaira-Hasegawa-like approximate likelihood ratio test (SH-aLRT) [40], and branch support was assessed using Ultrafast Bootstrap Approximation (UFB) [41] using 1000 bootstrap replicates for each method. FigTree v.1.4.4 (http://tree.bio.ed.ac.uk/software/figtree/) was used to visualize ML trees (Additional file 20: Figure S8). For phylogenetic analyses of oat and its diploid wild species, the plastome sequences of 25 published *Avena* species were downloaded from NCBI [24]. ML tree was constructed using above procedure (Additional file 20: Figure S10).

## Supplementary information

**Additional file 1: Table S1.** List of *Avena* species and their accession numbers in NCBI (or Genome Warehouse) included in the phylogenetic analyses of complete chloroplast genomes.

**Additional file 2: Table S2.** Characteristics of chloroplast genomes of analysed *Avena* species.

**Additional file 3: Table S3.** Nucleotide diversity (Pi) analyses of 13 *Avena* species (two congeneric species download from NCBI) and *Triticum aestivum*_NC002762.1 plastomes computed by DnaSP v.6 [29]. **a** Pi values of 131 plastid genes. The *rps12* is treated as three parts as the second gene divided into two independent transcription units. **b** Pi values of 130 intergenic regions.

**Additional file 4: Table S4.** Repetitive motif abundance in eleven *Avena* species and dandelion (*Taraxacum amplum*) plastomes computed by REPuter [30] and Phobos [31]. **a** Repetitive motif abundance computed by REPuter. F, P, R and C indicate the repeat types forward, palindrome, reverse and complement repeat, respectively. **b** Repeat type statistic analysis computed by REPuter. F, P, R and C indicate the repeat types forward, palindrome, reverse and complement, respectively. **c** Repeat distribution statistic analysis. LSC, SSC, IR, IGS and CDS indicate large single-copy, small single-copy, inverted repeat regions, intergenic and protein-coding sequences, respectively. **d** Tandem repeat abundance computed by Phobos. **e** Tandem repeat distribution statistic analysis.

**Additional file 5: Table S5.** Simple sequence repeat (SSR) sequences in eleven *Avena* species plastomes computed by Perlscript MicroSAtellite (MISA) [32]. **a** SSR sequence abundance. **b** SSR sequence distribution. IGS: intergenic sequences; CDS: protein-coding sequences; p1: mononucleotide repeat; p2: dinucleotide repeat; p3: trinucleotide repeat; p4: tetranucleotide repeat; c: composite SSR

**Additional file 6: Table S6.** Statistics between tandem repeats, indels and SNPs of plastome alignments between eleven *Avena* species presented here available in Genome Warehouse database and two *Avena* species available in NCBI (NC031650.1 and NC027468.1) with *A. sativa*_Liu312 as a reference. (**a**) Tandem repeats detected in *Avena sativa*_312 chloroplast genome using Phobos [31]. (**b**) Information of indels and SNPs in the complete chloroplast genome alignments.

**Additional file 7: Table S7.** The number and presence statistics of tandem repeats, indels and SNPs of plastome alignments between eleven *Avena* species presented here and two *Avena* species available in NCBI (NC031650.1 and NC027468.1) with *A. sativa*_Liu312 as a reference within each 150 bp window.

**Additional file 8: Table S8.** Maximum likelihood parameter estimates and substitutions for the 27 plastid genes (*atpA*, *atpE*, *atpF*, *atpI*, *ccsA*, *infA*, *matK*, *ndhA*, *ndhB*, *ndhD*, *ndhF*, *ndhH*, *ndhI*, *petA*, *psbA*, *rbcL*, *rpl2*, *rpl32*, *rpl33*, *rpoA*, *rpoB*, *rpoC1*, *rpoC2*, *rps2*, *rps3*, *rps11*, and *ycf4*).

**Additional file 9: Table S9.** GC percent and codon usage for *Avena* plastomes computed by CodonW v.1.4.2 [38]. **a** Genomic GC percent and codon usage statistic analysis. **b** The third codon usage statistic analysis.

**Additional file 10: Table S10.** Codon usage preference of all protein coding genes for eleven *Avena* plastomes calculated by http://www.bioinformatics.org/sms2/codon_usage. **a** Codon numbers of 20 amino acids and stop codons. **b** Codon number percent (%) of 20 amino acids and stop codons

**Additional file 11: Table S11.** The relative synonymous codon usage (RSCU) values for eleven *Avena* species computed by CodonW v.1.4.2 [38]. **a** RSCU values. **b** RSCU values subtracted by 1.00 (the expected value if no codon bias).

**Additional file 12: Figure S1.** Clean reads mapping to the assembled plastome of eleven *Avena* species. **a** *A. atlantica*. **b** *A. brevis*. **c** *A. eriantha*. **d** *A. hirtula*. **e** *A. longiglumis*. **f** *A. murphyi*. **g** *A. nuda*. **h** *A. sativa*. **i** *A. strigosa*. **j** *A. ventricosa*. **k** *A. wiestii*. The enrichment of *A. brevis*, *A. hirtula*, *A. strigosa* and *A. sativa* with PE250 bp reads from 300 bp insert libraries displays uniformity. It is better than those of the remaining seven species with PE250 bp reads from 500 bp insert libraries, whose reads enrichment displays valleys or peaks. There is no gap region along plastome sequences with the maximum coverage depth being 3004× to 9373 × .

**Additional file 13: Figure S2.** The intron sequence deletion of *rpoC1* gene of *Avena atlantica* (2049 bp; GWHAOPC01000000) and *Triticum urartu* (2052 bp; NC021762.1) compared to *Taraxacum amplum* (2746 bp; KX499525.1). **a** The 688 bp intron deletion marked by two black arrows in alignment sequences. **b-n** The *rpoC1* sequence alignment 1–2746 bp of *A. atlantica*, *Triticum urartu* and *Taraxacum amplum.*

**Additional file 14: Figure S3.** The *rpoC1* amino acid sequence alignments for *Avena* species, gramineous outgroups and *Taraxacum amplum* [11]. Amino acids that were conserved within *Avena* or among outgroup are colored according to physicochemical properties based on hydrophobicity color scheme. Black arrows indicated *Avena*-specific mutation.

**Additional file 15: Figure S4.** Comparison of border distance between adjacent genes and junctions of the LSC, SSC and two IR regions among (**a**) eleven *Avena* species and *Triticum aestivum* plastomes, and (**b**) fourteen *Avena* species plastomes available in NCBI. The adjacent border genes are denoted by colored boxes. The gaps between genes and the borders are denoted by the base pair (bp) lengths. The figure is not to scale with respect to sequence length and only shows relative changes near the IR/SC borders

**Additional file 16 Figure S5.** Repetitive motif abundance in eleven *Avena* and *Taraxacum amplum* plastomes (**a**) computed by REPuter [30]. F, P, R and C indicate the repeat types forward, palindrome, reverse and complement, respectively, and (**b**) computed by REPuter [30], to identify repeat sequences with length ≥ 21 bp and sequence identify ≥90%. **c** Tandem repeat distribution patterns by Phobos [31]. LSC, SSC, IR, IGS and CDS indicate large single-copy, small single-copy, inverted repeat regions, intergenic and protein-coding sequences, respectively.

**Additional file 17 Figure S6.** Visualization of simple sequence repeat (SSR) sequence distribution pattern in eleven *Avena* plastomes. IGS, CDS, c, p1 and p2 indicate intergenic regions, protein-coding sequences, composite SSR, mononucleotide repeat and dinucleotide repeat sequences, respectively

**Additional file 18 Figure S7.** Gene-specific Ka and Ka/Ks values between *Avena* plastomes. Ka, nonsynonymous rate; Ks, synonymous rate. Black solid dots denote gene-specific Ka/Ks values greater than 0.4000.

**Additional file 19 Figure S8.** Visualization of the relative synonymous codon usage (RSCU) [38] patterns in eleven *Avena* species. The RSCU values are subtracted by 1.00 (the expected value if no codon bias). The color scale indicates the magnitude of overall RSCU values: The greenest codons are the most preferred, and the reddest the least preferred. C/G-ending codons are in red, and A/T-ending codons are in blue.

**Additional file 20 Figure S9.** Maximum likelihood trees of 13 *Avena* species and *Triticum aestivum* based on the plastome matrix from two recombination hotspots, the combined ten most polymorphic genes, and ten most polymorphic intergenic regions. **a** ML tree based on combined LSC intermolecular recombination fragment sequences from *psbD* to *trnR*. **b** ML tree based on combined IRB intermolecular recombination fragment sequences from *ndhF* to *rps15*. **c** ML tree based on combined ten most polymorphic gene sequences (*rpl32*, *rpl16*, *psaC*, *psbF*, *ndhA*, *ndhC*, *atpF*, *matK*, *rpl22*, and *rps19*) of 13 *Avena* species. **d** ML tree based on combined ten most polymorphic intergenic sequences (*petG-trnW-CCA*, *ccsA-ndhD*, *rpl16-rps3*, *trnR-UCU-trnfM-CAU*, *rpl32-trnL-UAG*, *petB-petD*, *trnY-GUA-trnD-GUC*, *ndhE-ndhG*, *rps8-rpl14* and *psbH-petB*) of 13 *Avena* species. **e** ML tree based on combined ten most polymorphic gene sequences (*matK*, *rpl32*, *trnfM-CAU*, *trnK-UUU*, *rpl16*, *ndhA*, *psaC*, *psbF*, *ndhF* and *rpl22*) of 13 *Avena* species and *Triticum aestivum*. **f** ML tree based on combined ten most polymorphic intergenic sequences (*petG-trnW-CCA*, *ccsA-ndhD*, *rpl16-rps3*, *trnR-UCU-trnfM-CAU*, *rpl32-trnL-UAG*, *petB-petD*, *trnY-GUA-trnD-GUC*, *ndhE-ndhG*, *rps8-rpl14* and *psbH-petB*) of 13 *Avena* species and *Triticum aestivum*. Node numbers denote as: IQ-TREE [37] Shimodaira-Hasegawa test-approximate likelihood-ratio test support/Ultrafast bootstrap support (SH-aLRT support/UFBoot support) [38, 39].

**Additional file 21 Figure S10.** Maximum likelihood tree inferred from complete chloroplast genomes of eleven *Avena* species presented here available in Genome Warehouse database and 25 *Avena* species available in NCBI. *Triticum aestivum* is used as outgroup. Two clades are identified: clade I includes the *A. sativa* inserted subclade I (from *A. agadirianan* to *A. sterilis*), the *A. nuda* inserted subclade II (from *A. atlantica* to *A. nuda*) and the basal position of *A. longiglumis* together with extra three diploid species (*A. canariensis*, *A. damascene* and *A. lusitanica*), and clade II includes C-genome diploids. Node support denotes the maximum likelihood bootstrap value. Pink, red and green taxa correspond to A-, C-genome diploid and polyploid species in *Avena*, respectively.

## Data Availability

The complete chloroplast genomes generated in current study are released in the Genome Warehouse Database of National Genomics Data Center (https://bigd.big.ac.cn/search/?dbId=gwh&q=PRJCA003205) with accession numbers GWHAOPA01000000– GWHAOPK01000000 and in the NCBI database (https://www.ncbi.nlm.nih.gov/) with GenBank accession numbers MK336388–MK336398. The illumina datasets analysed during the current study are available in the Genome Sequence Archive (GSA) of National Genomics Data Center with accession number CRA003107 (https://bigd.big.ac.cn/search/?dbId=&q=CRA003107). All data generated or analysed during this study are included in this published article and the supplementary information files.

## Abbreviations

AIC: Akaike information criterion
BI: Bayesian inference
BLAST: Basic local alignment search tool
CDS: Protein-coding sequences
cp: Chloroplast
cpSSRs: Chloroplast simple sequence repeats
CTAB: Cetyl trimethylammonium bromide
C-terminal: Carboxy-terminal
DnaSP: DNA sequences polymorphism
GC: Guanine-cytosine
GTR: General time reversible
IGS: Intergenic sequences
Indel: Insertions/deletions
IR: Inverted repeat
IRA: Inverted repeat A
IRB: Inverted repeat B
IRs: Inverted repeat regions
Ka/Ks: Non-synonymous/synonymous mutation ratio
LSC: Large single copy
ML: Maximum likelihood
MLBS: Maximum likelihood bootstrap support
mRNAs: Messenger RNAs
NCBI: National Center for Biotechnology Information
N-terminal: Amino terminal
OGDRAW: OrganellarGenomeDRAW
Pi: Nucleotide diversity
rRNAs: Ribosomal RNAs
RSCU: Relative synonymous codon usage
SH-aLRT: Shimodaira-Hasegawa test-approximate likelihood-ratio test support
SNP: Single nucleotide polymorphism
SSC: Small single copy
SSRs: Simple sequence repeats
tRNAs: Transfer RNAs
UFBoot: Ultrafast bootstrap support
