# Supplementary material for: Comparative chloroplast genome analyses of Avena: insights into evolutionary dynamics and phylogeny
Source: BMC Plant Biol. 2020 Sep 2;20:406. doi: 10.1186/s12870-020-02621-y (PMC7466839; doi:10.1186/s12870-020-02621-y)
Supplement: Supplementary file 1 — Additional file 1: Table S1. List of Avena species and their accession numbers in NCBI (or Genome Warehouse) included in the phylogenetic analyses of complete chloroplast genomes. [file 12870_2020_2621_MOESM1_ESM.docx]

**Table S1**. List of *Avena* species and their accession numbers in GenBank (or Genome Warehouse) included in the phylogenetic analyses of complete chloroplast genomes.

| No. | Tribe | Taxa | Voucher source | Origin country | Length  (bp) | Gene | PCG | Accession no. in NCBI (or Genome Warehouse) |
| --- | --- | --- | --- | --- | --- | --- | --- | --- |
| 1 | Aveneae | *A*. *atlantica* B.R. Baum & Fedak (2*x =* 14; AA) | Liu 437 (PI 657294; IBSC) | Morocco: Ain Aouda | 135,940 | 131 | 84 | MK336394  (GWHAOPC01000000) |
| 2 |  | *A. brevis* Roth (2*x =* 14; AA) | Liu 289 (CN 1979; IBSC) | Canada: Ontario | 135,889 | 131 | 84 | MK336393  (GWHAOPA01000000) |
| 3 |  | *A*. *eriantha* Durieu (2*x =* 14; CpCp) | Liu 435 (PI 657575; IBSC) | Morocco: Ain Leuh | 135,909 | 131 | 84 | MK336395  (GWHAOPE01000000) |
| 4 |  | *A*. *hirtula* Lag. (2*x* = 14; AA) | Liu 299 (PI 657464; IBSC) | Morocco: Al Houceima | 135,937 | 131 | 84 | MK336392  (GWHAOPJ01000000) |
| 5 |  | *A*. *longiglumis* Durieu  (2*x* = 14; AA) | Liu 438 (PI 657389; IBSC) | Morocco: Moulay Bousselham | 135,962 | 131 | 84 | MK336391  (GWHAOPH01000000) |
| 6 |  | *A*. *murphyi* Ladiz. (4*x* = 28; AACC) | Liu 442 (PI 657355; IBSC) | Morocco: Tanger | 135,890 | 131 | 84 | MK336390  (GWHAOPF01000000) |
| 7 |  | *A*. *nuda* L. (6*x* = 42; AACCDD) | Liu 443 (CIav 9009; IBSC) | Canada: Ontario | 135,935 | 131 | 84 | MK336389  (GWHAOPD01000000) |
| 8 |  | *A*. *sativa* L. (6*x* = 42; AACCDD) | Liu 312 (PI 51385; IBSC) | Spain: Soria | 135,903 | 131 | 84 | MK336398  (GWHAOPK01000000) |
| 9 |  | *A*. *sativa* L.  (6*x* = 42; AACCDD) | *-* | *-* |  |  |  | NC027468.1 |
| 10 |  | *A*. *sterilis* L. (6*x* = 42; AACCDD) | *-* | - | 135,887 | 131 | 84 | NC031650.1 |
| 11 |  | *A*. *strigosa* Schreb.  (2*x* = 14; AA) | Liu 315 (CN 21993; IBSC) | Portugal: - | 135,935 | 131 | 84 | MK336397  (GWHAOPI01000000) |
| 12 |  | *A*. *ventricosa* Balansa ex Coss. (2*x* = 14; CvCv) | Liu 275 (PI 657337; IBSC) | Morocco: Ain Leuh | 135,910 | 131 | 84 | MK336396  (GWHAOPG01000000) |
| 13 |  | *A*. *wiestii* Steud. (2*x* = 14; AA) | Liu 439 (PI 657352; IBSC) | Morocco: Moulay Bousselham | 135,998 | 131 | 84 | MK336388  (GWHAOPB01000000) |
| 14 | Oryzeae | *Oryza sativa* L. (6*x* = 42; AACCDD) | *-* | - | 134,496 | 102 | 64 | NC008155.1 |
| 15 | Triticeae | *Aegilops speltoides* Tausch (6*x* = 42; AACCDD) | *-* | - | 113,536 | 111 | 77 | NC022135.1 |
| 16 |  | *Aegilops tauschii* Cosson (6*x* = 42; AACCDD) | *-* | - | 114,112 | 114 | 80 | NC022133.1 |
| 17 |  | *Hordeum vulgare* L. (6*x* = 42; AACCDD) | *-* | - | 136,462 | 141 | 83 | NC008590.1 |
| 18 |  | *Secale cereale* L. (6*x* = 42; AACCDD) | *-* | - | 114,843 | 111 | 77 | NC021761.1 |
| 19 |  | *Triticum aestivum* L. (6*x* = 42; AABBDD) | *-* | - | 134,545 | 137 | 83 | NC002762.1 |
| 20 |  | *Triticum uratu* Thumanjan ex Gandilyan (6*x* = 42; AACCDD) | *-* | - | 115,773 | 93 | 60 | NC021762.1 |
| 21 | Paniceae | *Cenchrus americanus* (L.) Morrone (6*x* = 42; AACCDD) | *-* | - | 138,172 | 130 | 83 | KX756179.1 |
| 22 | Andropogoneae | *Saccharum hybrid* (cultivar SP-80-3280) (6*x* = 42; AACCDD) | *-* | - | 141,182 | 144 | 97 | NC005878.1 |
| 23 |  | *Sorghum bicolor* (L.) Moench (6*x* = 42; AACCDD) | *-* | - | 140,754 | 140 | 84 | NC008602.1 |
| 24 | Andropogoneae | *Zea mays* L. (6*x* = 42; AACCDD) | *-* | - | 140,384 | 158 | 111 | NC001666.1 |
| 25 | Compositae | *Taraxacum amplum* Markl. (A978) (6*x* = 42; AACCDD) | *-* | - | 151,349 | 131 | 123 | KX499525.1 |

Taxa: Chromosome numbers based on http://mobot.mobot.org/W3T/Search/ipcn2.html; Genome assignment according to Liu et al. (2017). Voucher source: CN, Plant Gene Resources at Saskatchewan, Canada; PI or CIav, Germplasm Resources Information Network of United States Department of Agriculture at Beltsville, USA; IBSC, South China Botanical Garden Herbarium. -: unavailable; NCBI: National Center for Biotechnology Information; Genome Warehouse: Genome Warehouse Database in National Genomics Data Center.
