# Supplementary material for: Comparative chloroplast genome analyses of Avena: insights into evolutionary dynamics and phylogeny
Source: BMC Plant Biol. 2020 Sep 2;20:406. doi: 10.1186/s12870-020-02621-y (PMC7466839; doi:10.1186/s12870-020-02621-y)
Supplement: Supplementary file 2 — Additional file 2: Table S2. Characteristics of chloroplast genomes of analysed Avena species. [file 12870_2020_2621_MOESM2_ESM.docx]

**Table S2.** Characteristics of chloroplast genomes of analysed *Avena* species.

| Feature | *Avena atlantica* | *Avena brevis* | *Avena eriantha* | *Avena hirtula* | *Avena longiglumis* | *Avena murphyi* | *Avena nuda* | *Avena sativa* | *Avena strigosa* | *Avena ventricosa* | *Avena wiestii* |
| --- | --- | --- | --- | --- | --- | --- | --- | --- | --- | --- | --- |
| Size (bp) | 135,940 | 135,889 | 135,909 | 135,937 | 135,962 | 135,890 | 135,935 | 135,903 | 135,935 | 135,910 | 135,998 |
| LSC (bp) | 80,107 | 80,108 | 80,014 | 80,099 | 80,132 | 80,109 | 80,099 | 80,121 | 80,099 | 80,015 | 80,111 |
| SSC (bp) | 12,625 | 12,576 | 12,669 | 12,630 | 12,624 | 12,575 | 12,626 | 12,576 | 12,626 | 12,669 | 12,679 |
| IRs (bp) | 21,604 | 21,603 | 21,613 | 21,604 | 21,603 | 21,603 | 21,605 | 21,603 | 21,605 | 21,614 | 21,604 |
| Total genes | 131 | 131 | 131 | 131 | 131 | 131 | 131 | 131 | 131 | 131 | 131 |
| Protein-coding genes | 84 | 84 | 84 | 84 | 84 | 84 | 84 | 84 | 84 | 84 | 84 |
| tRNA | 39 | 39 | 39 | 39 | 39 | 39 | 39 | 39 | 39 | 39 | 39 |
| rRNA | 8 | 8 | 8 | 8 | 8 | 8 | 8 | 8 | 8 | 8 | 8 |
| Overall GC content (%) | 38.48 | 38.50 | 38.41 | 38.48 | 38.49 | 38.51 | 38.48 | 38.51 | 38.48 | 38.41 | 38.48 |
