# Supplementary figures and images for: Comparative chloroplast genome analyses of Avena: insights into evolutionary dynamics and phylogeny
Source: BMC Plant Biol. 2020 Sep 2;20:406. doi: 10.1186/s12870-020-02621-y (PMC7466839; doi:10.1186/s12870-020-02621-y)

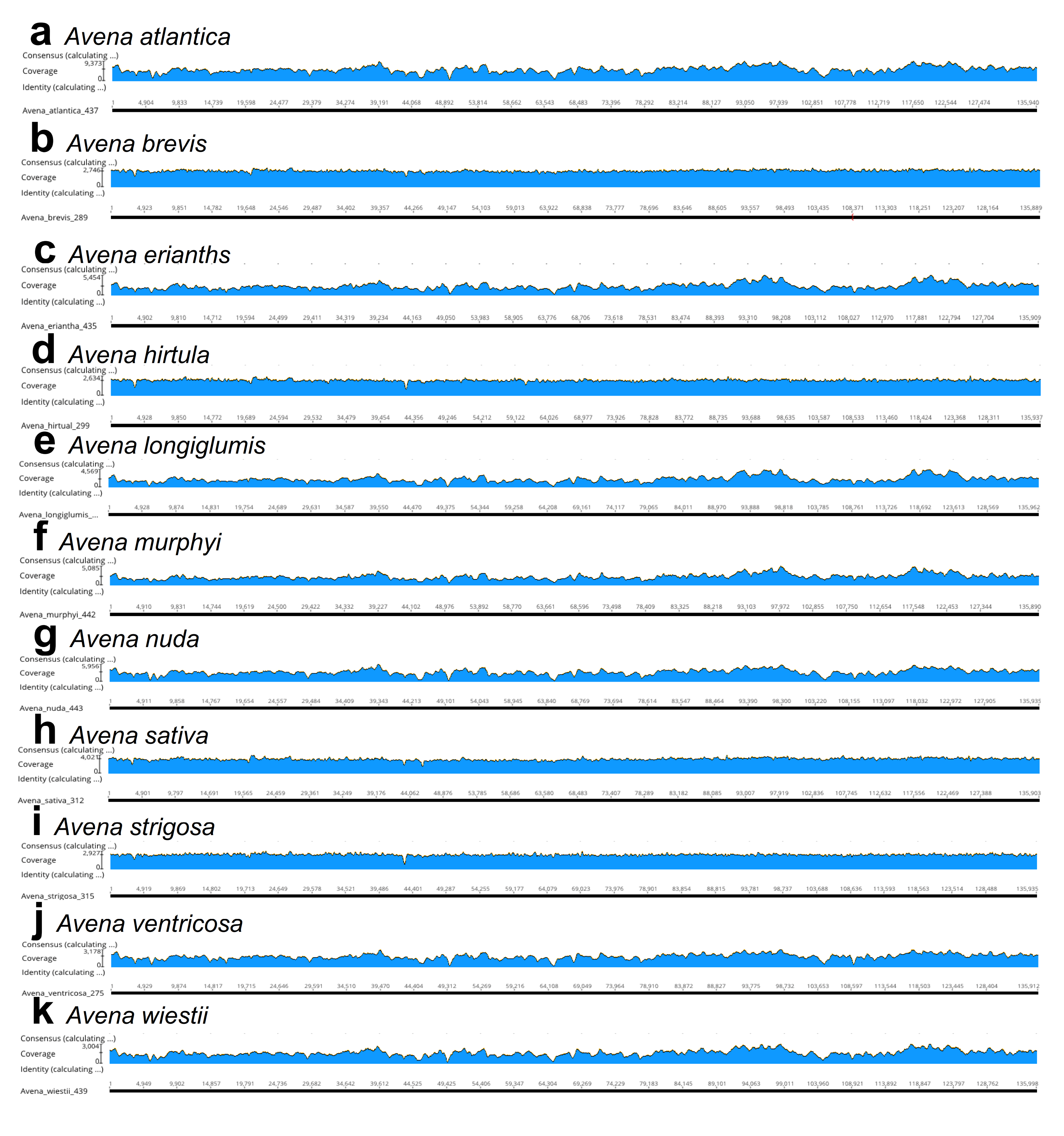

Supplement: Supplementary file 12 — Additional file 12: Figure S1. Clean reads mapping to the assembled plastome of eleven Avena species. a A. atlantica. b A. brevis. c A. eriantha. d A. hirtula. e A. longiglumis. f A. murphyi. g A. nuda. h A. sativa. i A. strigosa. j A. ventricosa. k A. wiestii. The enrichment of A. brevis, A. hirtula, A. strigosa and A. sativa with PE250 bp reads from 300 bp insert libraries displays uniformity. It is better than those of the remaining seven species with PE250 bp reads from 500 bp insert libraries, whose reads enrichment displays valleys or peaks. There is no gap region along plastome sequences with the maximum coverage depth being 3004× to 9373 × . [file 12870_2020_2621_MOESM12_ESM.tif]

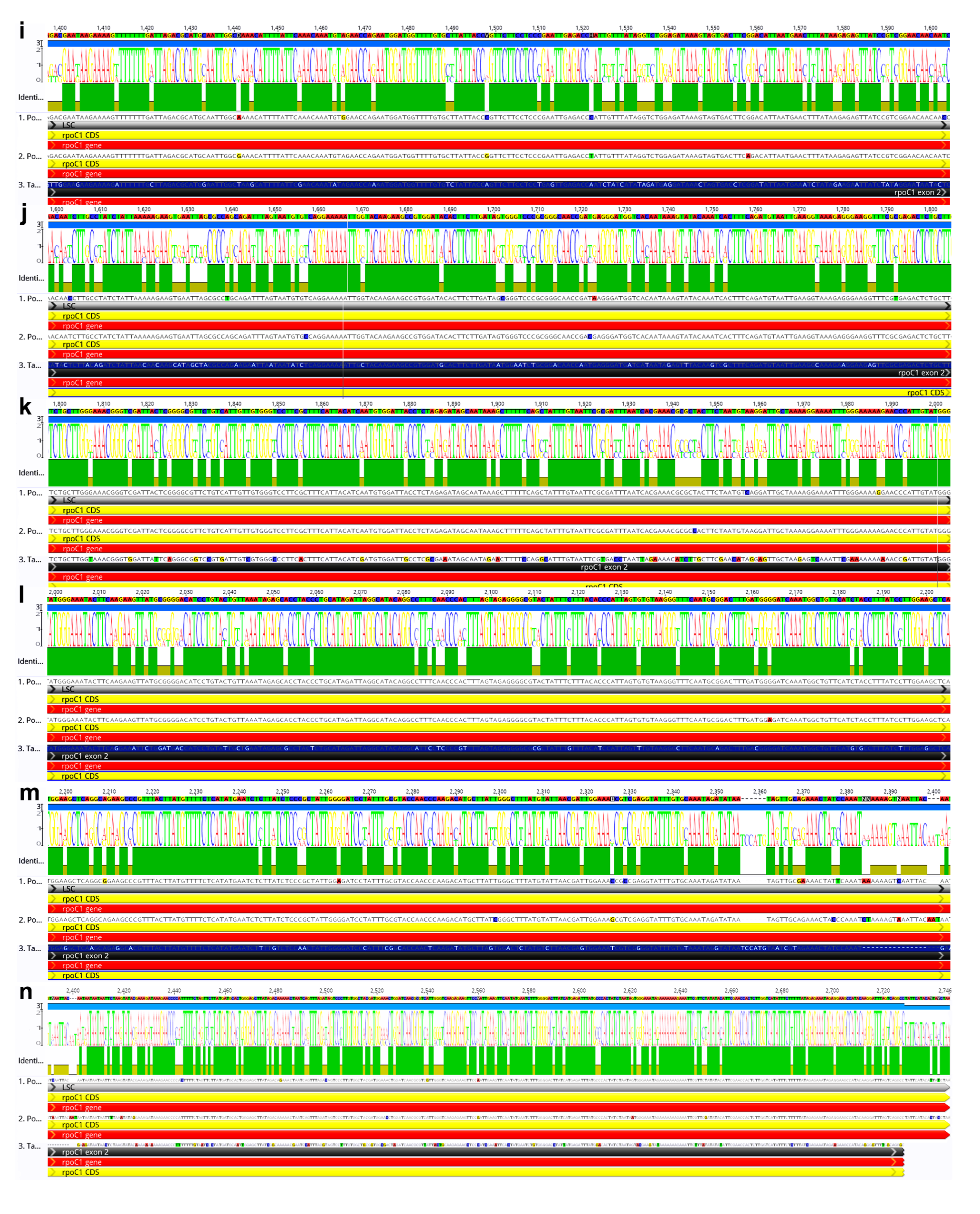

Supplement: Supplementary file 13 — Additional file 13: Figure S2. The intron sequence deletion of rpoC1 gene of Avena atlantica (2049 bp; GWHAOPC01000000) and Triticum urartu (2052 bp; NC021762.1) compared to Taraxacum amplum (2746 bp; KX499525.1). a The 688 bp intron deletion marked by two black arrows in alignment sequences. b-n The rpoC1 sequence alignment 1–2746 bp of A. atlantica, Triticum urartu and Taraxacum amplum. [file 12870_2020_2621_MOESM13_ESM.zip › Additional file13_Figure S2in.tif]

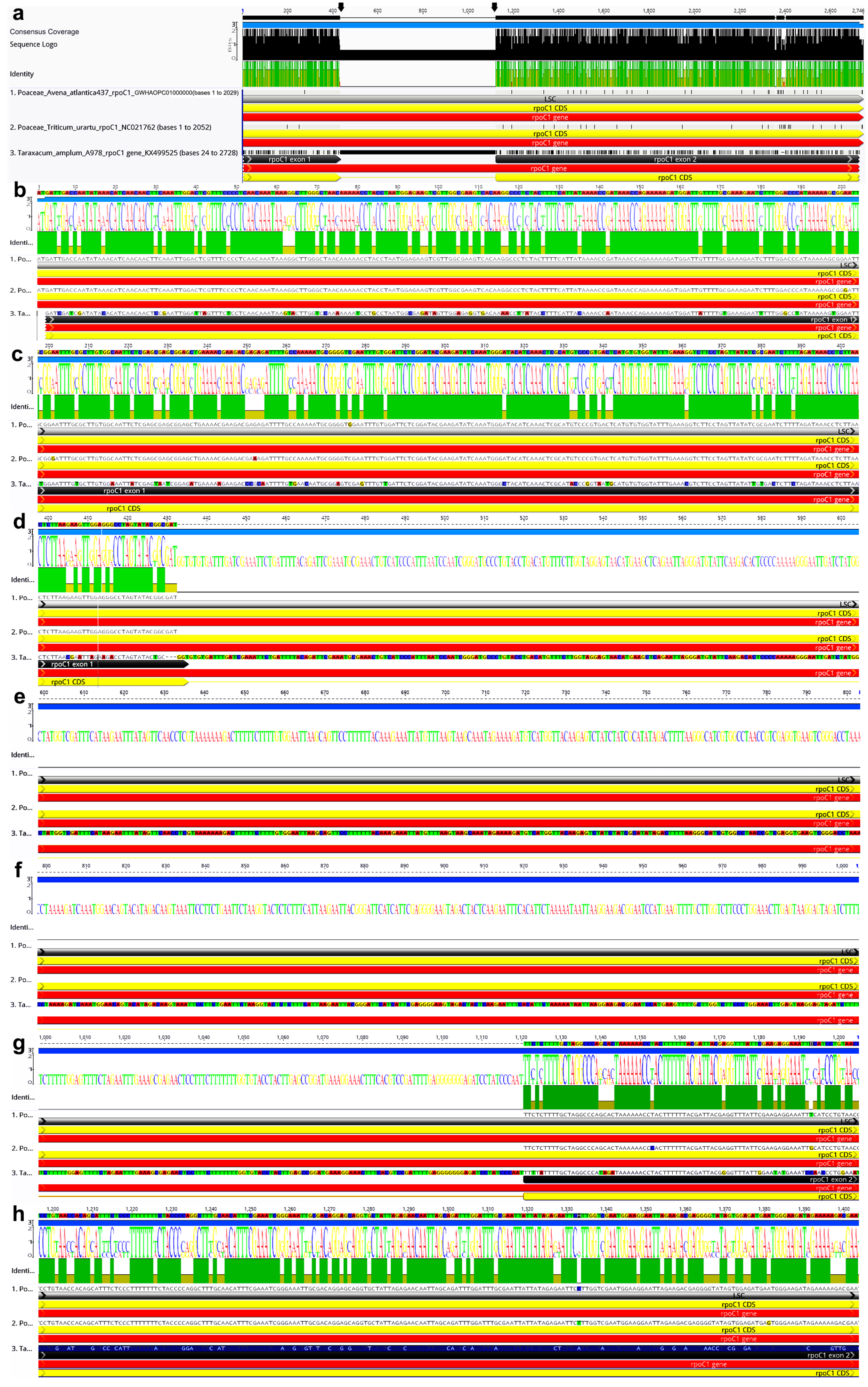

Supplement: Supplementary file 13 — Additional file 13: Figure S2. The intron sequence deletion of rpoC1 gene of Avena atlantica (2049 bp; GWHAOPC01000000) and Triticum urartu (2052 bp; NC021762.1) compared to Taraxacum amplum (2746 bp; KX499525.1). a The 688 bp intron deletion marked by two black arrows in alignment sequences. b-n The rpoC1 sequence alignment 1–2746 bp of A. atlantica, Triticum urartu and Taraxacum amplum. [file 12870_2020_2621_MOESM13_ESM.zip › Additional file13_Figure S2ah TIFF Tag.tif]

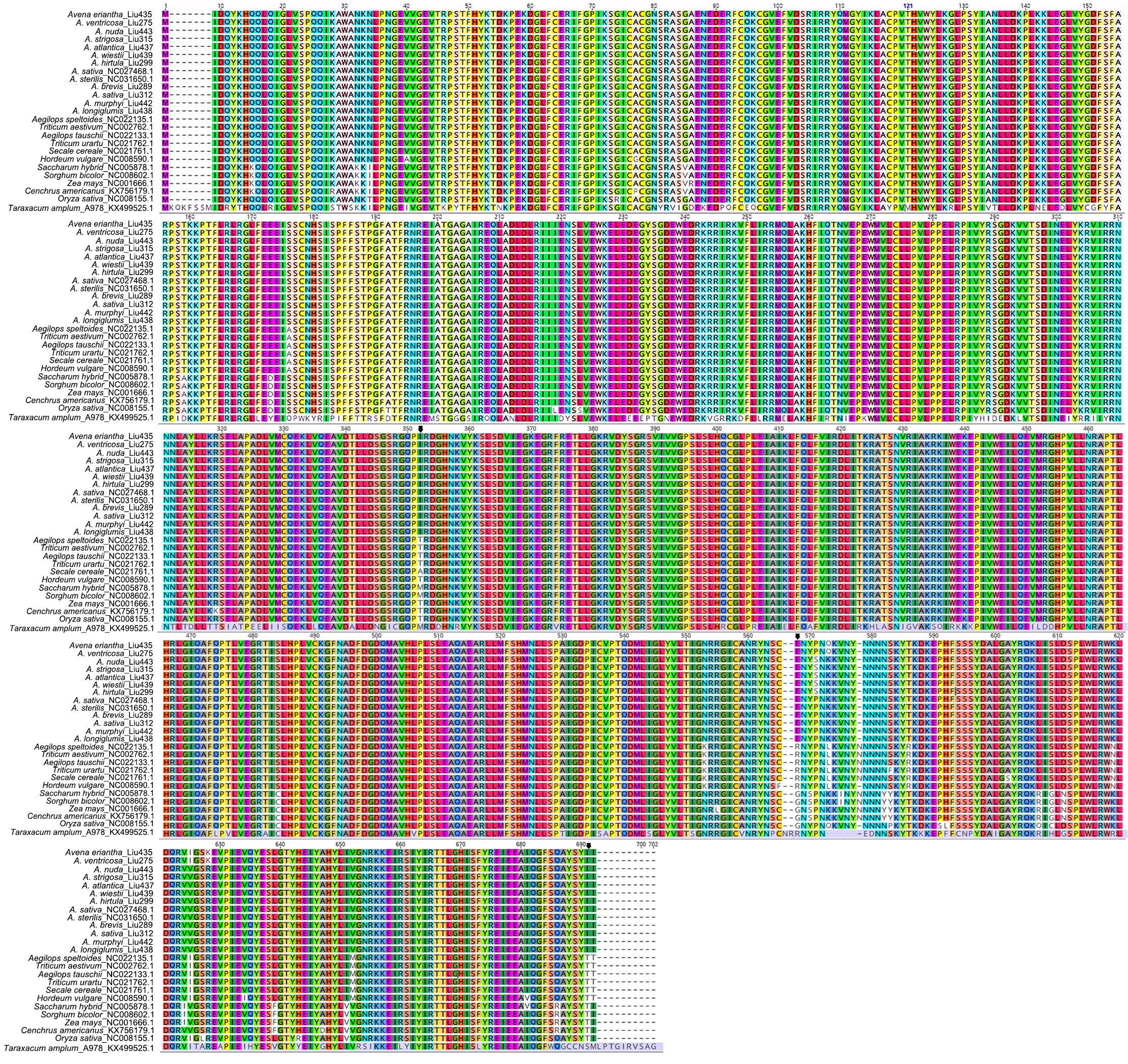

Supplement: Supplementary file 14 — Additional file 14: Figure S3. The rpoC1 amino acid sequence alignments for Avena species, gramineous outgroups and Taraxacum amplum [11]. Amino acids that were conserved within Avena or among outgroup are colored according to physicochemical properties based on hydrophobicity color scheme. Black arrows indicated Avena-specific mutation. [file 12870_2020_2621_MOESM14_ESM.tif]

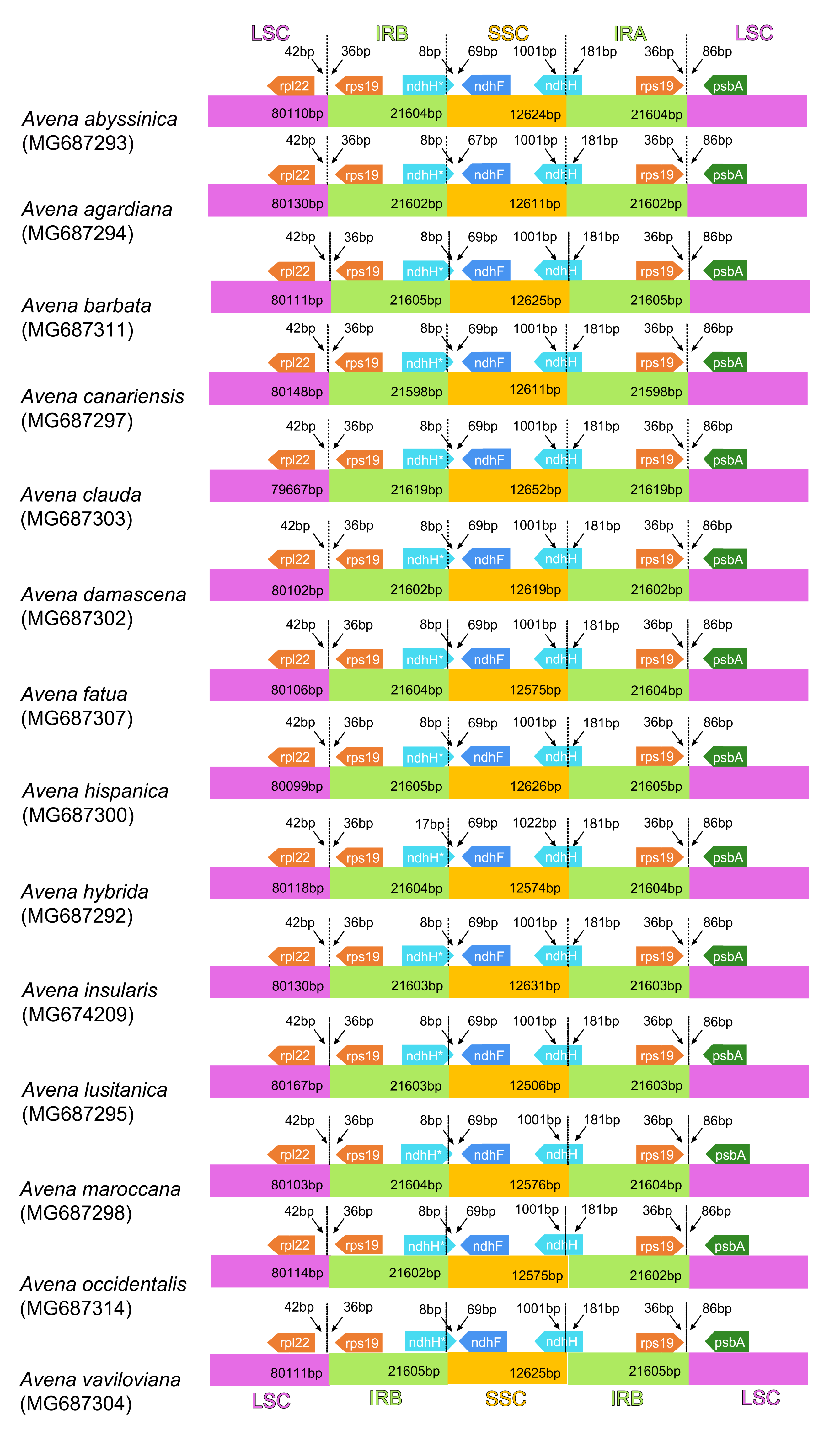

Supplement: Supplementary file 15 — Additional file 15: Figure S4. Comparison of border distance between adjacent genes and junctions of the LSC, SSC and two IR regions among (a) eleven Avena species and Triticum aestivum plastomes, and (b) fourteen Avena species plastomes available in NCBI. The adjacent border genes are denoted by colored boxes. The gaps between genes and the borders are denoted by the base pair (bp) lengths. The figure is not to scale with respect to sequence length and only shows relative changes near the IR/SC borders [file 12870_2020_2621_MOESM15_ESM.zip › Additional file15_Figure S4b.tif]

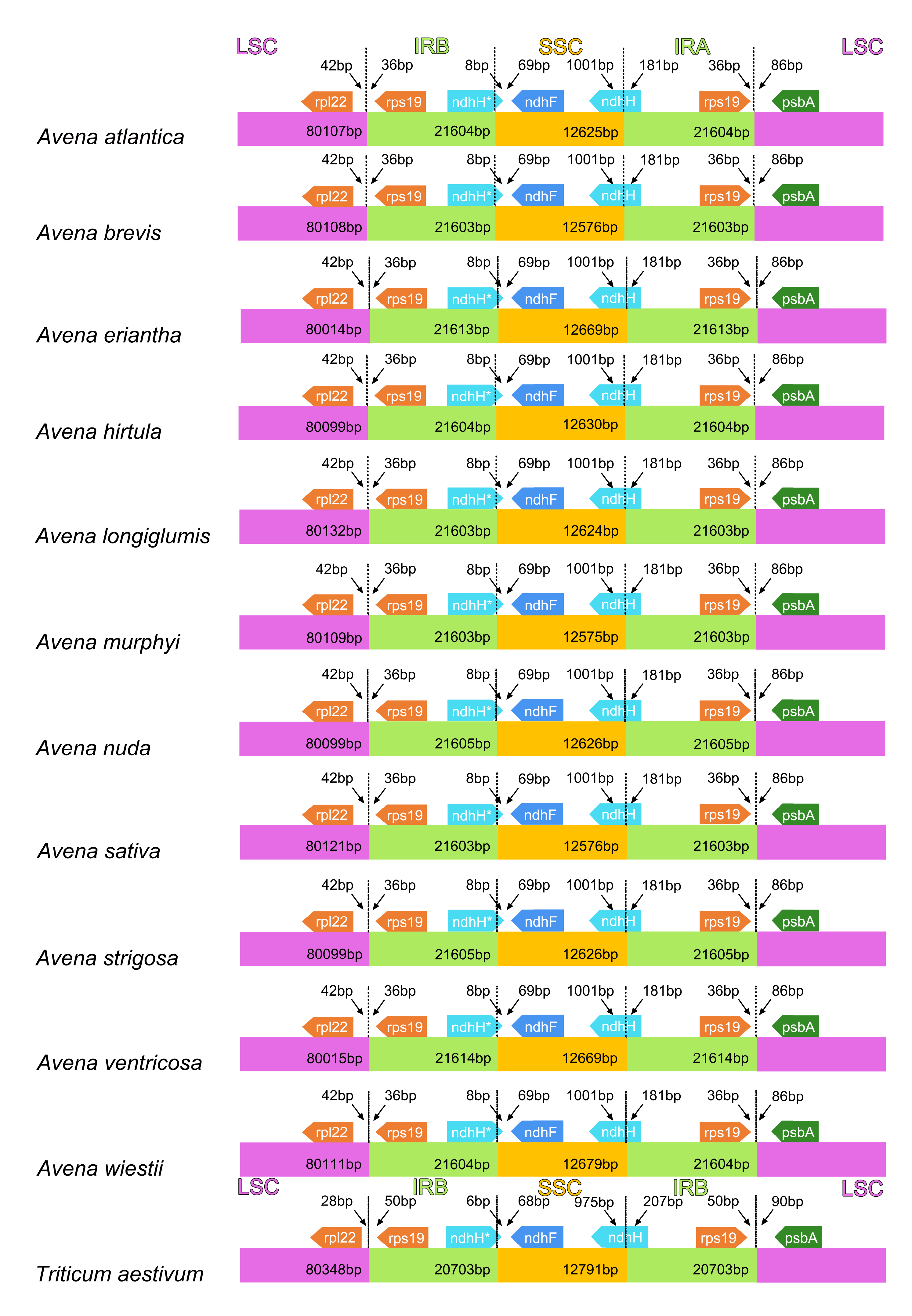

Supplement: Supplementary file 15 — Additional file 15: Figure S4. Comparison of border distance between adjacent genes and junctions of the LSC, SSC and two IR regions among (a) eleven Avena species and Triticum aestivum plastomes, and (b) fourteen Avena species plastomes available in NCBI. The adjacent border genes are denoted by colored boxes. The gaps between genes and the borders are denoted by the base pair (bp) lengths. The figure is not to scale with respect to sequence length and only shows relative changes near the IR/SC borders [file 12870_2020_2621_MOESM15_ESM.zip › Additional file15_Figure S4a.tif]

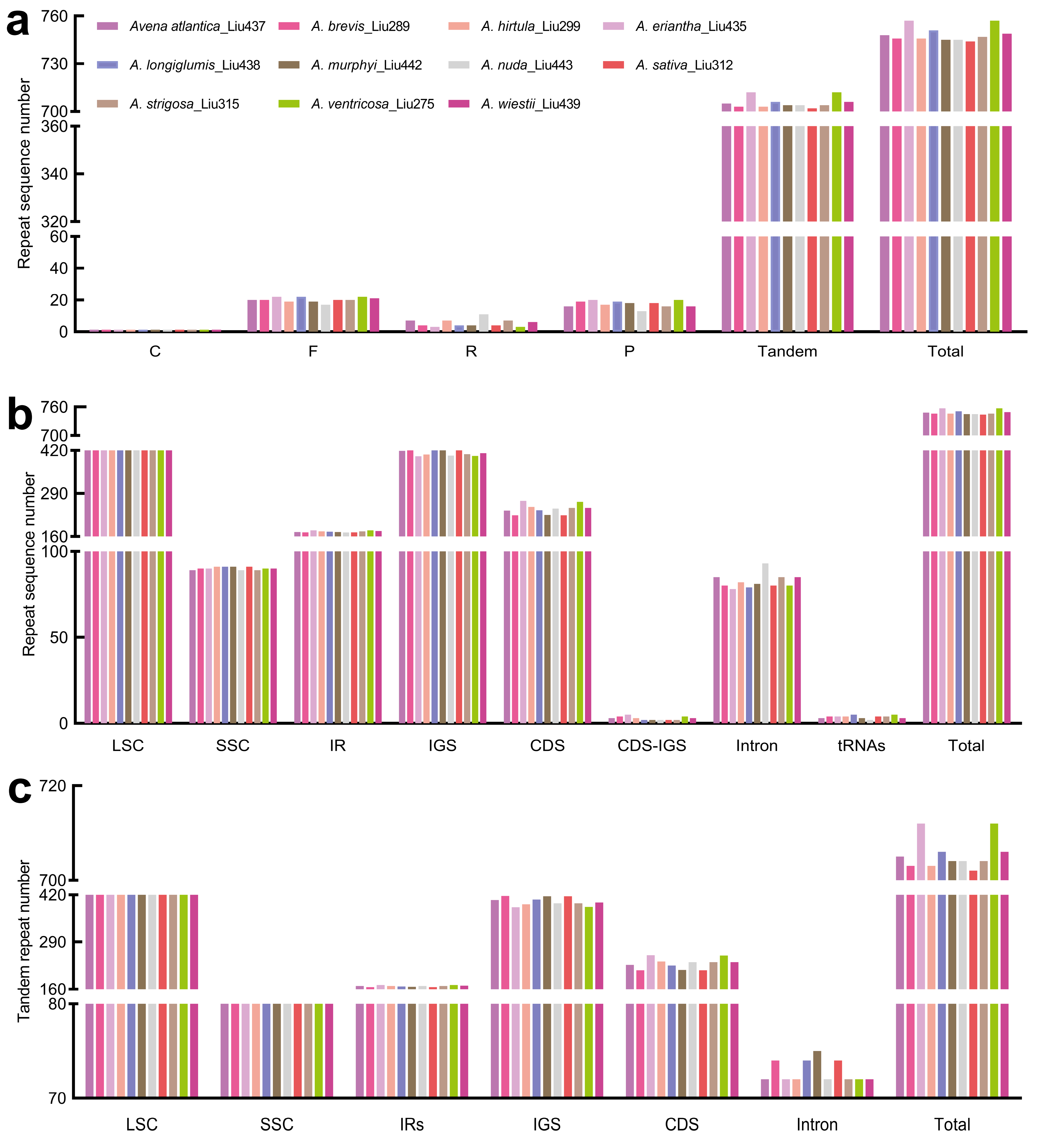

Supplement: Supplementary file 16 — Additional file 16 Figure S5. Repetitive motif abundance in eleven Avena and Taraxacum amplum plastomes (a) computed by REPuter [30]. F, P, R and C indicate the repeat types forward, palindrome, reverse and complement, respectively, and (b) computed by REPuter [30], to identify repeat sequences with length ≥ 21 bp and sequence identify ≥90%. c Tandem repeat distribution patterns by Phobos [31]. LSC, SSC, IR, IGS and CDS indicate large single-copy, small single-copy, inverted repeat regions, intergenic and protein-coding sequences, respectively. [file 12870_2020_2621_MOESM16_ESM.tif]

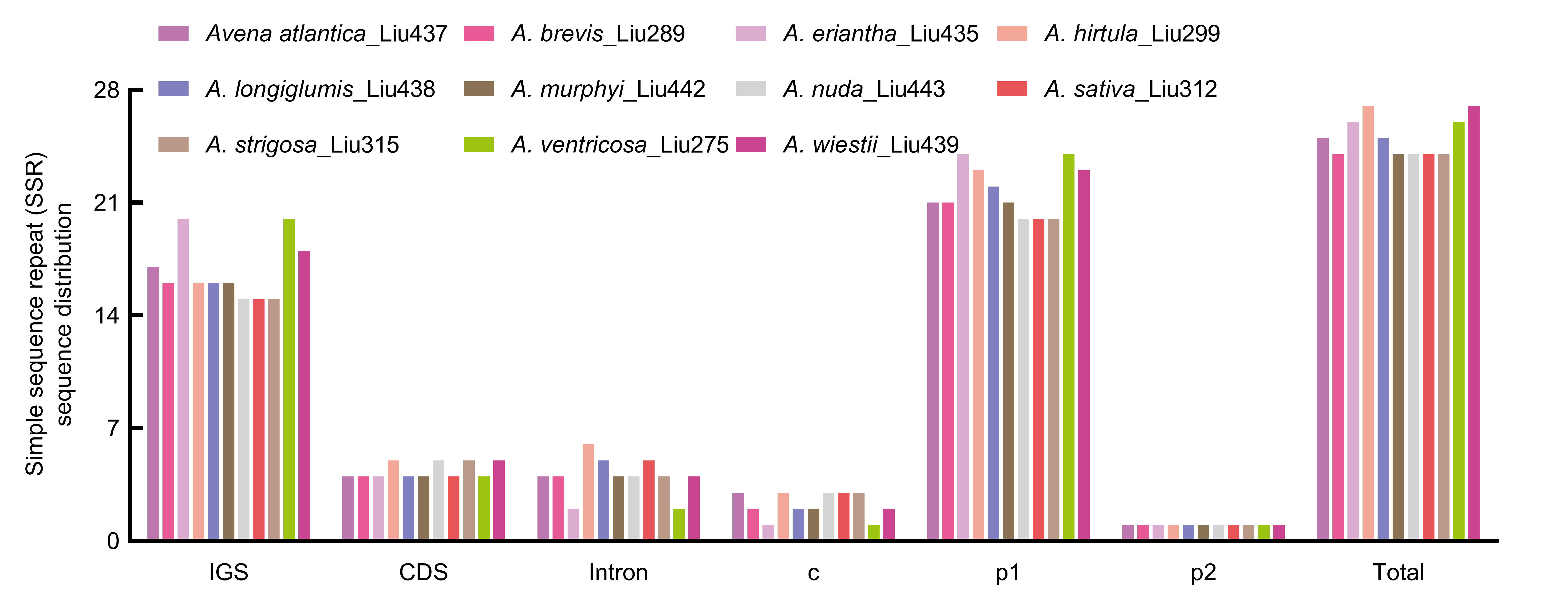

Supplement: Supplementary file 17 — Additional file 17 Figure S6. Visualization of simple sequence repeat (SSR) sequence distribution pattern in eleven Avena plastomes. IGS, CDS, c, p1 and p2 indicate intergenic regions, protein-coding sequences, composite SSR, mononucleotide repeat and dinucleotide repeat sequences, respectively [file 12870_2020_2621_MOESM17_ESM.tif]

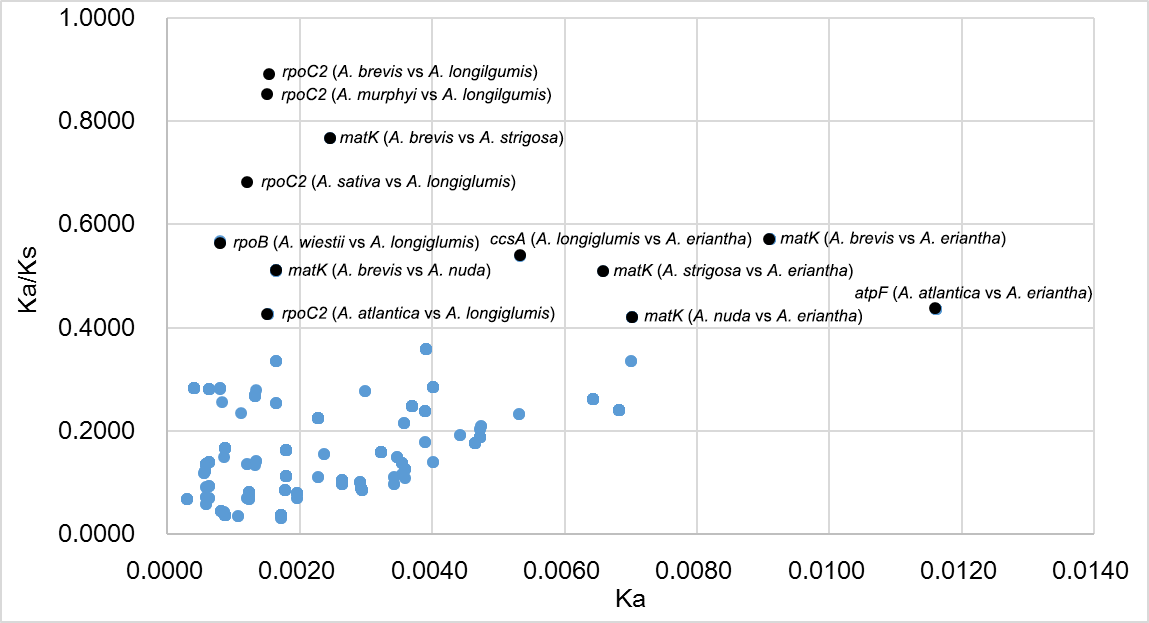

Supplement: Supplementary file 18 — Additional file 18 Figure S7. Gene-specific Ka and Ka/Ks values between Avena plastomes. Ka, nonsynonymous rate; Ks, synonymous rate. Black solid dots denote gene-specific Ka/Ks values greater than 0.4000. [file 12870_2020_2621_MOESM18_ESM.tif]

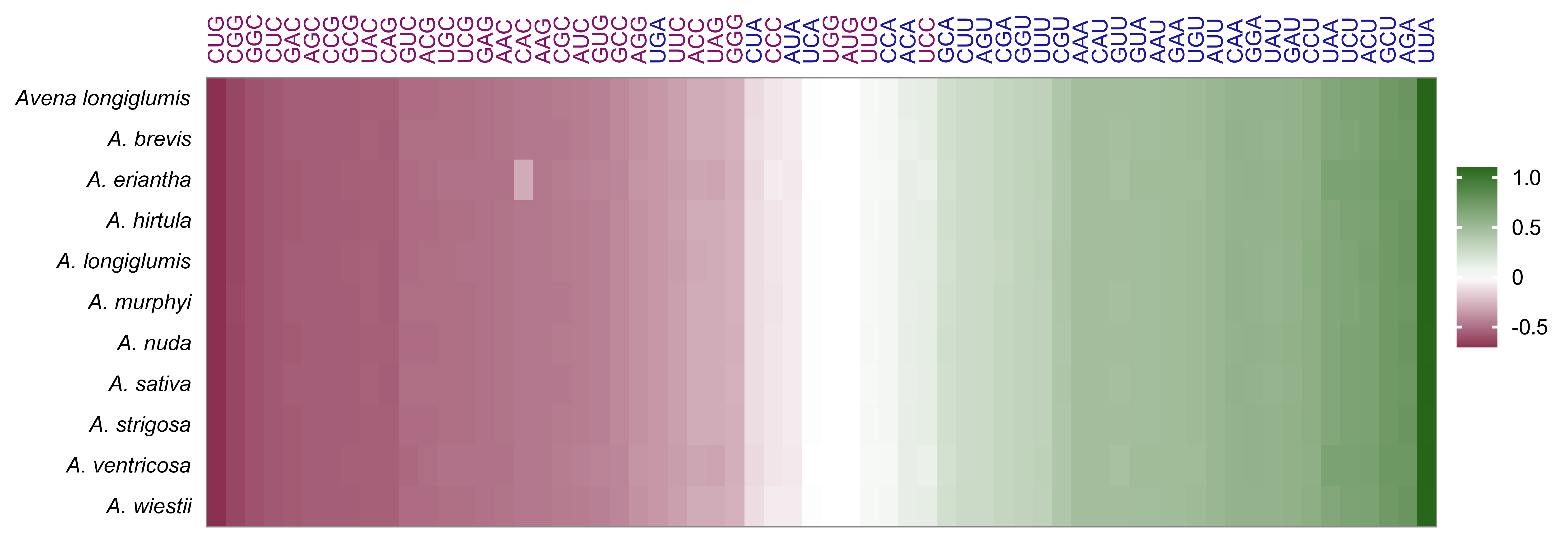

Supplement: Supplementary file 19 — Additional file 19 Figure S8. Visualization of the relative synonymous codon usage (RSCU) [38] patterns in eleven Avena species. The RSCU values are subtracted by 1.00 (the expected value if no codon bias). The color scale indicates the magnitude of overall RSCU values: The greenest codons are the most preferred, and the reddest the least preferred. C/G-ending codons are in red, and A/T-ending codons are in blue. [file 12870_2020_2621_MOESM19_ESM.tif]

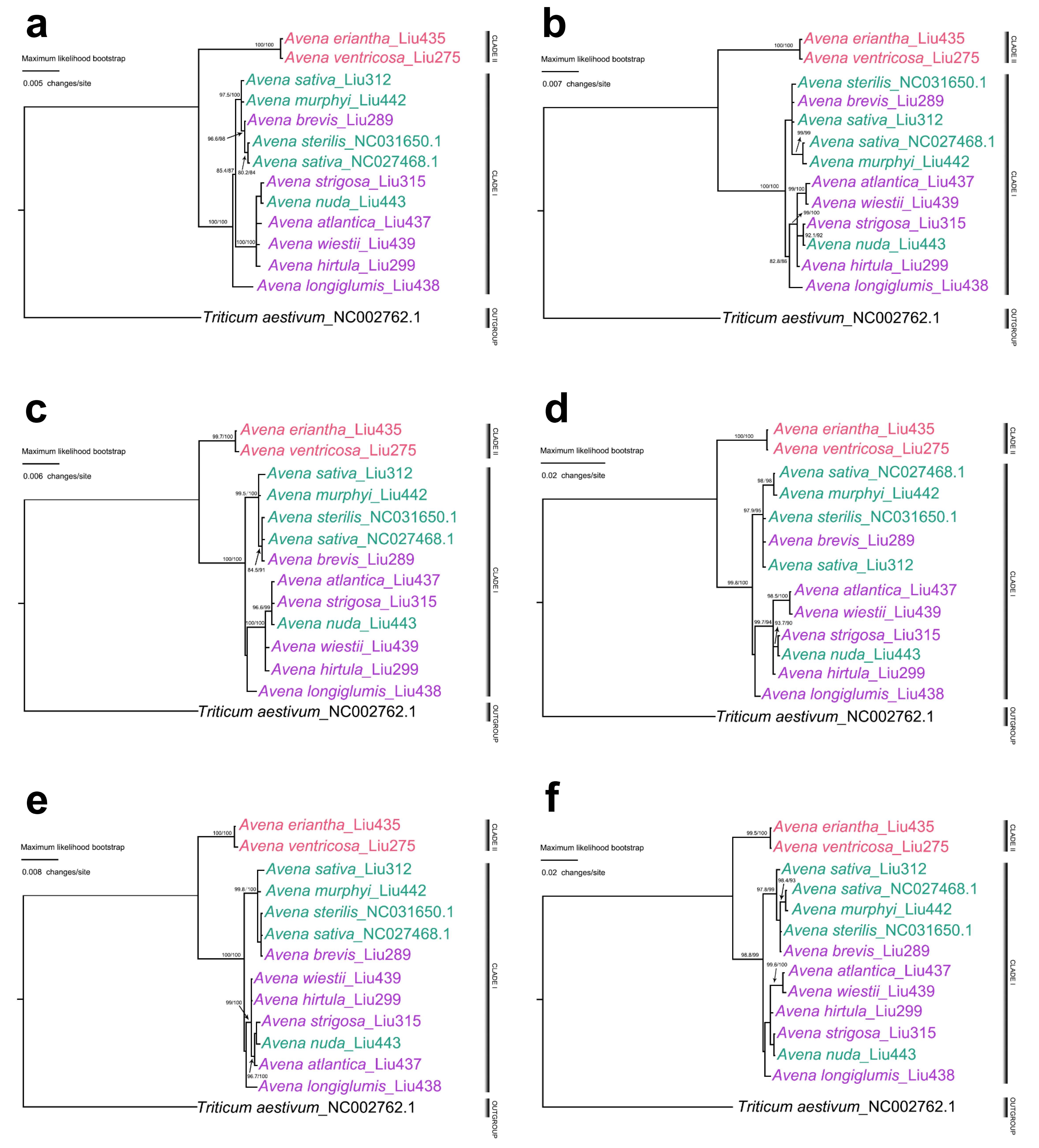

Supplement: Supplementary file 20 — Additional file 20 Figure S9. Maximum likelihood trees of 13 Avena species and Triticum aestivum based on the plastome matrix from two recombination hotspots, the combined ten most polymorphic genes, and ten most polymorphic intergenic regions. a ML tree based on combined LSC intermolecular recombination fragment sequences from psbD to trnR. b ML tree based on combined IRB intermolecular recombination fragment sequences from ndhF to rps15. c ML tree based on combined ten most polymorphic gene sequences (rpl32, rpl16, psaC, psbF, ndhA, ndhC, atpF, matK, rpl22, and rps19) of 13 Avena species. d ML tree based on combined ten most polymorphic intergenic sequences (petG-trnW-CCA, ccsA-ndhD, rpl16-rps3, trnR-UCU-trnfM-CAU, rpl32-trnL-UAG, petB-petD, trnY-GUA-trnD-GUC, ndhE-ndhG, rps8-rpl14 and psbH-petB) of 13 Avena species. e ML tree based on combined ten most polymorphic gene sequences (matK, rpl32, trnfM-CAU, trnK-UUU, rpl16, ndhA, psaC, psbF, ndhF and rpl22) of 13 Avena species and Triticum aestivum. f ML tree based on combined ten most polymorphic intergenic sequences (petG-trnW-CCA, ccsA-ndhD, rpl16-rps3, trnR-UCU-trnfM-CAU, rpl32-trnL-UAG, petB-petD, trnY-GUA-trnD-GUC, ndhE-ndhG, rps8-rpl14 and psbH-petB) of 13 Avena species and Triticum aestivum. Node numbers denote as: IQ-TREE [37] Shimodaira-Hasegawa test-approximate likelihood-ratio test support/Ultrafast bootstrap support (SH-aLRT support/UFBoot support) [38, 39]. [file 12870_2020_2621_MOESM20_ESM.tif]

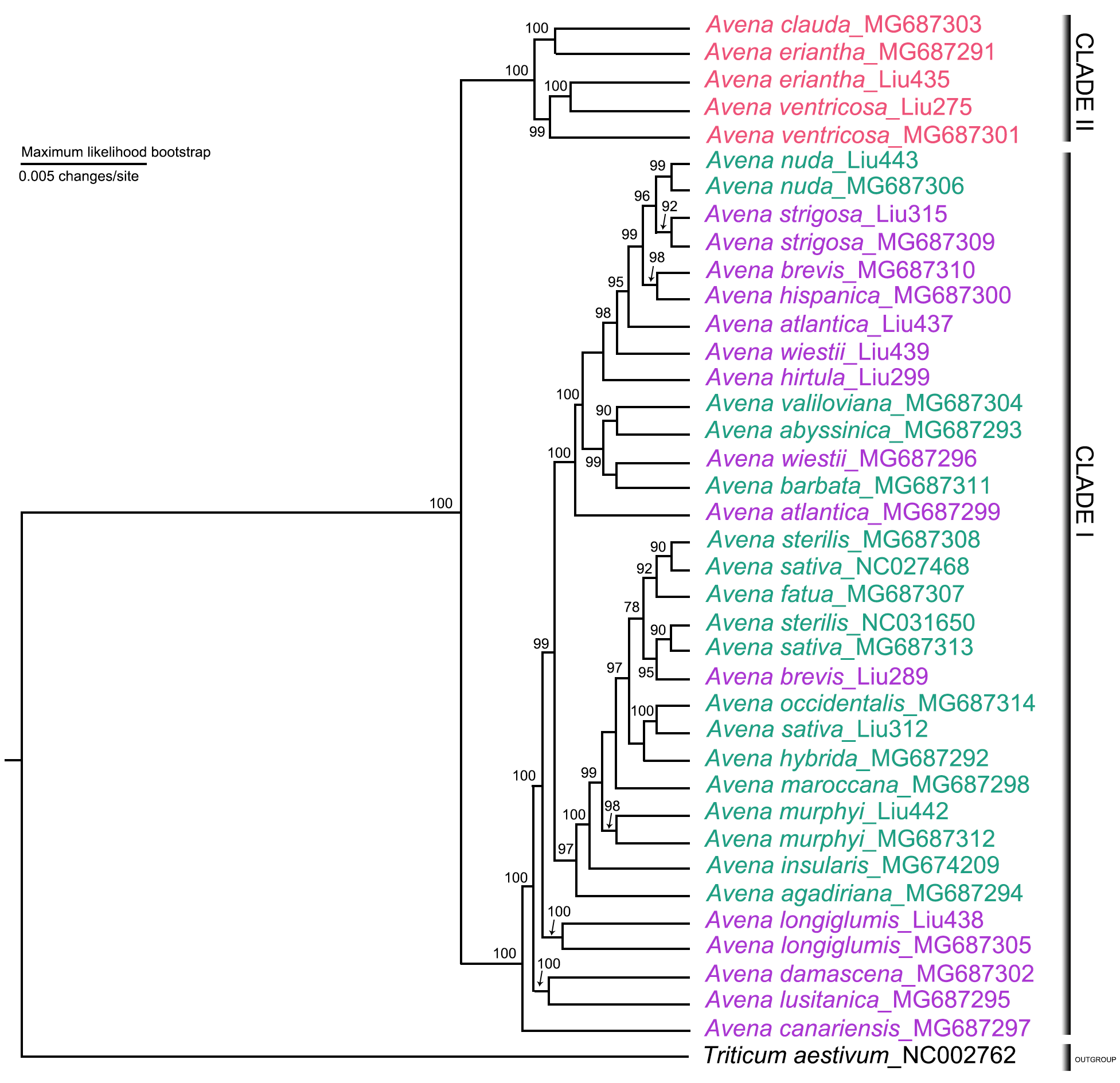

Supplement: Supplementary file 21 — Additional file 21 Figure S10. Maximum likelihood tree inferred from complete chloroplast genomes of eleven Avena species presented here available in Genome Warehouse database and 25 Avena species available in NCBI. Triticum aestivum is used as outgroup. Two clades are identified: clade I includes the A. sativa inserted subclade I (from A. agadirianan to A. sterilis), the A. nuda inserted subclade II (from A. atlantica to A. nuda) and the basal position of A. longiglumis together with extra three diploid species (A. canariensis, A. damascene and A. lusitanica), and clade II includes C-genome diploids. Node support denotes the maximum likelihood bootstrap value. Pink, red and green taxa correspond to A-, C-genome diploid and polyploid species in Avena, respectively. [file 12870_2020_2621_MOESM21_ESM.tif]
